# Relationships of maternal and paternal anthropometry with neonatal body size, proportions and adiposity in an Australian cohort

**DOI:** 10.1002/ajpa.22680

**Published:** 2014-12-13

**Authors:** Emma Pomeroy, Jonathan CK Wells, Tim J Cole, Michael O'Callaghan, Jay T Stock

**Affiliations:** 1 Newnham College, University of CambridgeCambridge, UK; 2 Division of Biological Anthropology, Department of Archaeology and Anthropology, University of CambridgeCambridge, UK; 3 Childhood Nutrition Research Centre, UCL Institute of Child HealthLondon, UK; 4 Population Policy and Practice Programme, UCL Institute of Child HealthLondon, UK; 5 School of Medicine, Mater Clinical School, University of QueenslandBrisbane, Australia

**Keywords:** neonatal anthropometry, birthweight, limb length, parental height, parental BMI

## Abstract

The patterns of association between maternal or paternal and neonatal phenotype may offer insight into how neonatal characteristics are shaped by evolutionary processes, such as conflicting parental interests in fetal investment and obstetric constraints. Paternal interests are theoretically served by maximizing fetal growth, and maternal interests by managing investment in current and future offspring, but whether paternal and maternal influences act on different components of overall size is unknown. We tested whether parents' prepregnancy height and body mass index (BMI) were related to neonatal anthropometry (birthweight, head circumference, absolute and proportional limb segment and trunk lengths, subcutaneous fat) among 1,041 Australian neonates using stepwise linear regression. Maternal and paternal height and maternal BMI were associated with birthweight. Paternal height related to offspring forearm and lower leg lengths, maternal height and BMI to neonatal head circumference, and maternal BMI to offspring adiposity. Principal components analysis identified three components of variability reflecting neonatal “head and trunk skeletal size,” “adiposity,” and “limb lengths.” Regression analyses of the component scores supported the associations of head and trunk size or adiposity with maternal anthropometry, and limb lengths with paternal anthropometry. Our results suggest that while neonatal fatness reflects environmental conditions (maternal physiology), head circumference and limb and trunk lengths show differing associations with parental anthropometry. These patterns may reflect genetics, parental imprinting and environmental influences in a manner consistent with parental conflicts of interest. Paternal height may relate to neonatal limb length as a means of increasing fetal growth without exacerbating the risk of obstetric complications. Am J Phys Anthropol 156:625–636, 2015.

Fetal growth and development have important implications across the life-course, influencing the risk of birth complications (Koyanagi et al., 2013), neonatal morbidity and mortality (Karn and Penrose, 1951; McIntire et al., 1999), the schedule and trajectory of postnatal growth (Smith et al., 1976; Mei et al., 2004), reproductive function (Lummaa, 2003) and adult disease risk (Hales and Barker, 1992; Barker, 1998). Given the extensive implications of early growth and development, we might expect the prenatal period to be an important stage at which parental genetic, epigenetic or phenotypic factors may influence offspring phenotype. Understanding these influences on fetal growth may offer insights into the evolutionary processes affecting early development.

The genotype and phenotype of both parents are associated with fetal and neonatal phenotype (Lindsay et al., 2000; Hyppönen et al., 2003; Anderson et al., 2006; Carone et al., 2010; Ng et al., 2010; Myklestad et al., 2012; Hillman et al., 2013; Tyrrell et al., 2013; Wells et al., 2013; Wei et al., 2014). From an evolutionary perspective, parents may have conflicting “interests” in early offspring growth (Haig and Westoby, 1989; Moore and Haig, 1991). As the mother provides all the prenatal physiological investment, her lifetime reproductive success will be maximized by balancing investment in current and future offspring, since she will be equally related to each of them. In contrast, the father's interests are best served by maximizing maternal investment in the current offspring, since her prior and/or subsequent offspring may not be his. Parental genes may therefore be involved in a “tug-of-war” over maternal resources, with paternal genes promoting and maternal genes constraining fetal growth (Haig and Westoby, 1989; Moore and Haig, 1991).

Studies of humans and using animal models suggest that parental genes influence different aspects of placental size and physiology to promote (paternal) or restrict (maternal) fetal growth in a manner consistent with parental conflict theory (Willison, 1991; Allen et al., [Bibr b4],[Bibr b5]; Hitchins and Moore, 2002; Apostolidou et al., 2007; Demetriou et al., 2014). For example, expression levels of paternally expressed genes (e.g., *IGF2*) are positively associated and those of maternally expressed genes (e.g., *PHLDA2*) negatively associated with birthweight (reviewed in Ishida and Moore, 2013).

Other constraints are also likely to influence fetal development, such as maternal obstetric dimensions (reviewed in Rosenberg and Trevathan, 2002; Wells et al., 2012; Pomeroy et al., In press). Environmentally responsive aspects of maternal phenotype including height and pelvic geometry (Liselele et al., 2000; Kjærgaard et al., 2010; Benjamin et al., 2012), and neonatal characteristics including head and shoulder dimensions (Trevathan and Rosenberg, 2000; Rosenberg and Trevathan, 2002), likely contribute to the risk of obstructed labour resulting from a mismatch between fetal size and maternal pelvic dimensions. Associations between grandmaternal malnutrition and newborn size, and secular increases in birthweight, suggest that fetal development is “tailored” to current maternal pelvic dimensions to avoid such obstetric complications (Pembrey, 1996). While fathers lose potential reproductive success if the offspring and mother die through obstructed labor, the penalty in lifetime reproductive success is much greater for the mother if she dies in childbirth, creating further tension between maternal and paternal interests in fetal growth.

It is unknown whether the outcome of this parental “tug of war” may also lead to differing associations between parental phenotype and distinct components of fetal growth, but detailed analyses of neonatal phenotype (limb, trunk and head size, adiposity) may offer insight into this question. We therefore examined associations of maternal and paternal anthropometry [height and body mass index (BMI)] with offspring characteristics including birthweight, head circumference, absolute and proportional limb segment and trunk lengths, and skinfolds. We hypothesized that maternal and paternal anthropometry would show differing associations with different components of neonatal phenotype.

## MATERIALS AND METHODS

**Figure 1:**
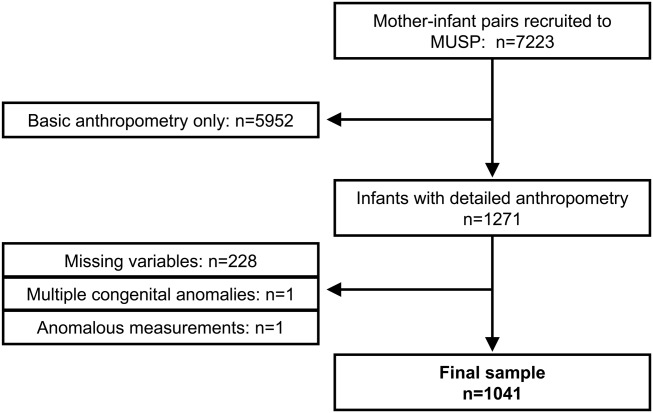
Flow chart describing composition of the study sample and its relationship to the full Mater-University of Queensland Study of Pregnancy (MUSP) dataset.

We analyzed data on neonatal and parental anthropometry from the Mater-University of Queensland Study of Pregnancy (MUSP) dataset (Najman et al., 2005). The study was approved by ethics committees from the Mater Hospitals and the University of Queensland, and maternal oral informed consent was obtained (in keeping with standards at the time of this phase of the study in the early 1980s). The study recruited 7,223 mother–infant pairs in Brisbane, Australia, although detailed anthropometry that included limb segment lengths was only collected in the subset of neonates (*n* = 1271 live singleton births, 668 males) born between 1982 and 1983, on which our analysis focuses. For the present analyses, the dataset was further limited to individuals with complete anthropometry and explanatory variables, and two infants with anomalous measurements or multiple congenital anomalies were also omitted, leaving a total sample of 1,041 neonates (Fig. 1).

McGrath et al. (2005) reported that there were no significant differences in birthweight or sex ratio between the full sample and the sample for which detailed anthropometry were recorded, except a small difference in gestational age that was statistically, but unlikely to be biologically, significant (0.1 weeks longer among included neonates; *P* <0.01). Maternally reported ethnicity of the parents in the sample was overwhelmingly “White” (91% of 1,216 mothers and 93% of 1,167 fathers on whom data were available, remaining parents split approximately equally between “Asian” and “Aboriginal/Islander”).

All babies were measured by a trained research nurse (Keeping, 1981; McGrath et al., 2005) within 24 h of birth. No data on inter-rater reliability are available. The neonatal measurements in this analysis were: birthweight; head, abdominal, upper arm, lower arm, thigh and lower leg circumferences; face, biparietal, shoulder and hip breadths; neck-rump, upper arm, forearm, thigh, and lower leg lengths; and subscapular, triceps, abdominal and anterior thigh skinfolds. Data were confirmed graphically to follow a normal distribution.

Maternal height (to nearest cm) was measured at the first prenatal clinic visit, while paternal height (to nearest cm) and weight, and maternal prepregnancy weight (to nearest kg), were self-reported. They were used to calculate parental BMI, and natural logarithms of parental height and BMI formed the primary explanatory variables. Sex and gestational age (e.g., Catalano et al., 1995; Hindmarsh et al., 2002; Knight et al., 2005; Shields et al., 2006) and several potential confounding variables (maternal smoking, education, parity: Kramer et al., 2000; Raum et al., 2001; Voigt et al., 2004; Harvey et al., 2007; Elshibly and Schmalisch, 2009; Jansen et al., 2009; van den Berg et al., 2013) were included in the analyses based on associations reported in the literature. Potential confounders were recorded at the first clinic visit or extracted from medical records. Parity was coded as 0 vs. 1 or more. Maternal education was coded into dummy variables for three categories: incomplete- (reference), complete-, and post-high school. Maternal smoking in the last trimester was coded as yes or no, and maternal age at birth in years was also recorded. Data were available on family income but were omitted from analyses as they were not significant in the regression models.

Multiple regression was used to analyze the relationship between neonatal body measurements (as dependent variables) and parental height and BMI, adjusted for the potential confounding variables specified in the Results. Dependent variables were natural logarithms of head circumference, neck-rump length, upper arm length, forearm length, thigh length, lower leg length, birthweight, and sum of 4 skinfolds (subscapular, triceps, abdominal, and anterior thigh), as well as the following limb proportions calculated from the log transformed data: relative upper (upper arm length + forearm length, adjusting for neck-rump length in the regression model) and lower limb lengths (thigh length + lower leg length, adjusting for neck-rump length); and intralimb indices: brachial (forearm length adjusting for upper arm length in the regression model) and crural index (lower leg length adjusting for thigh length). Neonatal measurements were selected to represent diverse aspects of neonatal phenotype, including fatness and head, trunk and limb dimensions. The proportions of limb to trunk lengths were calculated to further highlight any differing relationships between the different body segments and parental anthropometry that may exist. The relative lengths of the distal (forearm or lower leg) to proximal (upper arm or thigh) limb bones were calculated since distal limb segment lengths may be particularly sensitive to environmental growth disturbance (Meadows Jantz and Jantz, 1999; Lampl et al., 2003; Bailey et al., 2007; Pomeroy et al., [Bibr b97],[Bibr b98]), but the relationship between neonatal intralimb proportions and parental anthropometry is unknown.

Male sex, gestational age (weeks) and potential confounders were entered in the first round of the regression model where *P* < 0.1. Parental heights and BMIs were entered in the second round using a stepwise procedure, with *P* < 0.01 (rather than 0.05 due to the number of analyses performed). Interaction terms between offspring sex and parental anthropometry variables were also tested for. Where the equivalent anthropometry of both parents was significant in the model (e.g., both parents' heights), we ran an otherwise identical regression model where parental heights were replaced with log geometric mean and log ratio of the two parents' heights. The significance of the log ratio term indicates the significance of the difference in maternal and paternal regression coefficients, and *P* < 0.01 was considered significant due to multiple analyses.

To further explore the relationships between maternal, paternal and neonatal anthropometry, all available neonatal measurements were submitted to principal components analysis (PCA) with varimax rotation to maximize the distinction between components and facilitate interpretation (Kaiser, 1958). PCA reduces the variables to a smaller set of variables, or principal components (PCs), which are linear combinations of the original variables that explain the majority of the variance in those variables (Dunteman, 1989). Each of the component scores for the first three PCs was analyzed with multiple regression as described for the original data. PCA was performed on pooled sex data since initial analyses (not shown) demonstrated little sex difference. Analyses were performed using SPSS version 21.0 for Windows.

## RESULTS

The characteristics of the study sample are summarized in Tables1 and 2. Five hundred and forty nine neonates were male (53%), and mean birthweights of males and females were 3.52 kg and 3.40 kg, very close to the medians (3.38 kg and 3.26 kg for males and females, respectively) from recent international standards (Villar et al., 2014). Seven of the 1,043 babies (0.7%) were of low birthweight (i.e., <2.5 kg). Mean height and BMI were 163 cm and 22.0 kg/m^2^ for the mothers and 176 cm and 23.6 kg/m^2^ for the fathers. Forty-one percent were first births, 37% of mothers smoked, and mean maternal age at the child's birth was 25.8 years.

**Table 1:** Neonatal characteristics of the study sample

Characteristic	Female		Male		Combined	
	Mean	SD	Mean	SD	Mean	SD
Birth weight (g)	3399	450	3521	430	3463	440
Head circumference (mm)	348	12	355	12	352	12
Biparietal diameter (mm)	94	3.5	95	3.6	95	3.6
Face diameter (mm)	86	4.1	87	4.3	86	4.3
Neck-rump length (mm)	227	15	229	14	228	15
Shoulders width (mm)	157	9.8	159	11	158	10
Hips width (mm)	133	10	134	11	133	11
Upper arm length (mm)	83	6.6	85	6.9	84	6.8
Upper arm circumference (mm)	109	9.2	110	9.0	110	9.1
Lower arm length (mm)	60	8.2	62	7.9	61	8.1
Lower arm circumference (mm)	100	7.7	101	7.2	100	7.4
Chest circumference (mm)	333	17	335	17	334	17
Abdomen circumference (mm)	289	20	288	17	288	19
Thigh length (mm)	89	6.8	90	6.7	90	6.8
Thigh circumference (mm)	155	14	154	13	155	14
Lower leg length (mm)	68	7.9	70	8.1	69	8.0
Lower leg circumference (mm)	113	8.6	113	8.3	113	8.4
Skinfold subscapular (mm)	55	10	52	10	54	10
Skinfold abdominal (mm)	35	6.0	35	6.3	35	6
Skinfold triceps (mm)	50	9.1	49	8.8	49	9.0
Skinfold anterior thigh (mm)	67	14	63	14	65	14
Gestational age (weeks)	40	1.2	40	1.3	40	1.2

**Table 2:** Parental characteristics of the study sample

*Continuous variables*	Offspring sex					
	Female (n=492)		Male (n=549)		Combined (n=1041)	
	Mean	SD	Mean	SD	Mean	SD
Maternal height (cm)	163	6.3	163	6.2	163	6.2
Maternal BMI (kg/m^2^)	21.8	3.7	22.1	4.2	22.0	4.0
Paternal height (cm)	177	7.9	176	7.9	176	7.9
Paternal BMI (kg/m^2^)	23.5	3.4	23.6	3.7	23.6	3.5
Maternal age (years)	25.7	4.9	25.8	5.1	25.8	5.0

*Categorical variables*	n(%)	n (%)	n (%)
Maternal education			
Incomplete high school	89 (18)	91 (17)	180 (17)
Complete high school	320 (65)	359 (65)	679 (65)
Post-high school	83 (17)	99 (18)	182 (18)
Maternal smoking			
No	300 (61)	353 (64)	653 (63)
Yes	192 (39)	196 (36)	388 (37)
Parity			
0	194 (39)	231 (42)	425 (41)
1+	298 (61)	318 (58)	616 (59)

**Figure 2:**
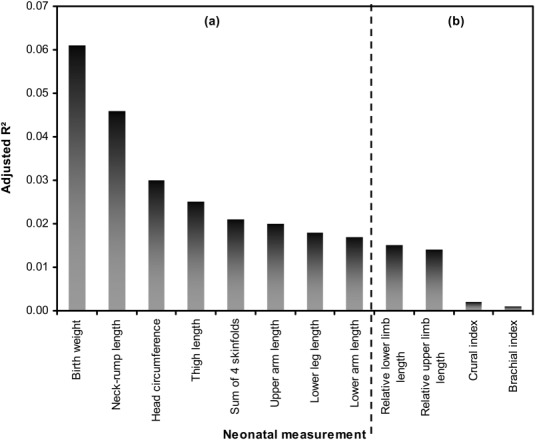
Adjusted *R*^2^ values for variation in neonatal anthropometry explained by parental anthropometry. (a) Absolute measurements; (b) limb proportion indices.

**Figure 3:**
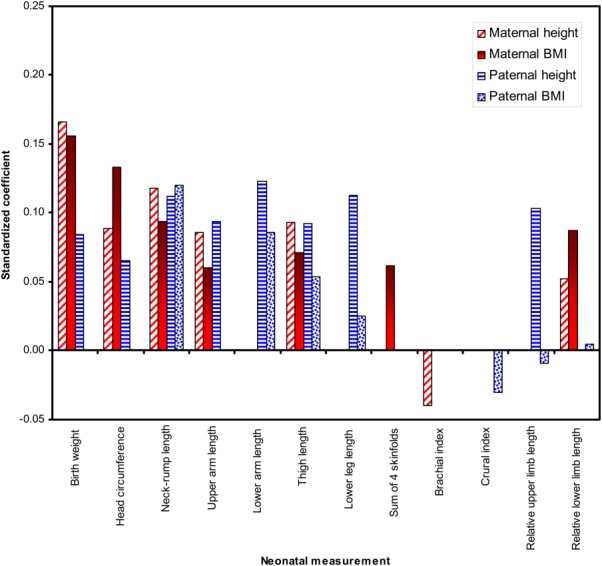
Standardized coefficients (*β*) for variation in neonatal anthropometry explained by parental anthropometry. [Color figure can be viewed in the online issue, which is available at wileyonlinelibrary.com.]

The adjusted *R*^2^ values for the final regression models (Fig. 2 and Table3) indicated that adjusting for potential confounders (see Supporting Information Table2 for details of confounders in each model), parental anthropometry explained a small proportion of variance in neonatal anthropometry. Parental measurements explained the greatest amount of variation in birthweight (6%) and neck-rump length (5%), but less variance in head circumference (3%), summed skinfolds (2%), limb segment lengths (2%), and limb proportion indices (0–1%). Birthweight was significantly associated with maternal height and BMI and paternal height (Fig. 3 and Table3). Associations were twice as strong for maternal vs. paternal height, but not statistically different (*P* = 0.03). Neck-rump length related similarly to both parents' heights and BMIs, with no significant differences between parental height or BMI coefficients (*P* > 0.1). Head circumference related to maternal height and BMI only, and the sum of four skinfolds was only associated with maternal BMI. Proximal limb segment lengths (upper arm, thigh) related equally strongly to paternal and maternal height (*P* > 0.1 in all tests for differences in parental height coefficients). In addition, maternal BMI was significantly associated with neonatal thigh length. Distal limb segments (lower arm, lower leg) were associated only with paternal anthropometry (both height and BMI). Limb: trunk length indices were associated with paternal height only, and intralimb indices did not relate to parental anthropometry. Offspring sex by parental anthropometry interactions were excluded from the models as they were not significant.

**Table 3:** Final regression models of neonatal anthropometry on parental anthropometry, adjusting for potential confounders

Measurement	Maternal height		Maternal BMI		Paternal height		Paternal BMI		Adjusted *R*^2^
	*β*	*P*	*β*	*P*	*β*	*P*	*β*	*P*	
Birth weight	0.17	<0.001	0.16	<0.001	0.08	0.003			0.06
Neck-rump length	0.12	<0.001	0.09	0.002	0.11	<0.001	0.12	0.008	0.05
Head circumference	0.10	<0.001	0.14	<0.001					0.03
Sum of 4 skinfolds			0.14	<0.001					0.02
Upper arm length	0.08	0.007			0.10	0.001			0.02
Lower arm length					0.12	<0.001	0.09	0.006	0.02
Thigh length	0.10	0.001	0.08	0.008	0.08	0.008			0.02
Lower leg length					0.12	<0.001	0.09	0.003	0.02
Relative upper limb length					0.10	0.002			0.01
Relative lower limb length					0.09	0.004			0.01
“Brachial index”									0.00
“Crural index”									0.00

All variables log transformed.

The PCA analysis showed the same general patterns. Three PCs were derived using varimax rotation (Table4). PC1 represented “head and trunk skeletal size,” as head circumference and breadths were most strongly loaded, followed by shoulder and hip widths and birthweight. PC2 represented “adiposity,” as skinfold thicknesses had the highest loadings, followed by limb circumferences (which reflect both adipose and lean tissue). PC3 represented “limb lengths.” Multiple regression analysis indicated significant positive associations between PC1 (“head and trunk skeletal size”) and male offspring sex, gestational age, and maternal education, height and BMI, and a negative relationship with maternal smoking (Table5). PC2 scores (“adiposity”) related positively to gestational age, parity and maternal BMI and were lower among sons. PC3 (“limb lengths”) was positively associated with male offspring sex, gestational age, and paternal height and BMI (Table3), and negatively with maternal smoking.

**Table 4:** Variable loadings for the first three principal components from principal components analysis of neonatal anthropometry

Measurement	Unrotated component			Varimax rotated component		
	1	2	3	1	2	3
Birth weight	**0.93**	−0.04	0.11	**0.69**	0.52	0.38
Head circumference	**0.74**	−0.05	0.39	**0.76**	0.20	0.29
Biparietal width	**0.60**	−0.24	0.54	**0.84**	0.05	0.07
Face width	0.49	−0.52	0.50	**0.83**	0.08	−0.23
Neck-rump length	0.57	−0.03	0.26	0.55	0.18	0.24
Shoulder width	**0.71**	−0.24	0.19	**0.67**	0.37	0.11
Hip width	**0.63**	−0.36	0.18	**0.65**	0.37	−0.04
Upper arm length	0.57	**0.63**	0.07	0.19	0.14	**0.82**
MUAC	**0.86**	0.00	−0.09	0.49	**0.60**	0.37
Lower arm length	0.42	**0.78**	−0.02	−0.01	0.07	**0.89**
Lower arm circumference	**0.88**	−0.03	−0.09	0.51	**0.62**	0.36
Chest circumference	**0.83**	0.11	0.06	0.54	0.45	0.47
Abdomen circumference	**0.82**	0.12	−0.06	0.44	0.52	0.46
Thigh length	0.57	**0.66**	0.11	0.21	0.10	**0.84**
Thigh circumference	**0.82**	−0.12	−0.10	0.51	**0.62**	0.24
Lower leg length	0.41	**0.79**	0.02	0.00	0.03	**0.89**
Lower leg circumference	**0.88**	−0.05	−0.10	0.51	**0.64**	0.33
Subscapular skinfold	**0.65**	−0.24	−0.52	0.15	**0.85**	0.05
Abdominal skinfold	**0.61**	0.11	−0.47	0.03	**0.69**	0.34
Triceps skinfold	0.53	−0.35	−0.46	0.15	**0.76**	−0.10
Anterior thigh skinfold	**0.65**	−0.38	−0.41	0.27	**0.82**	−0.08
Variance explained (%)	47.8	14.1	8.3	25.3	24.3	20.6

Bold indicates loadings ≥|0.6|.

**Table 5:** Regression analysis of principal component (PC) scores from neonatal anthropometry on parental anthropometry and potential confounding variables

Principal Component	Model term	Standardized coefficient (β)	*P*
PC1: head and trunk skeletal size	(Constant)		<0.001
	Male sex	0.21	<0.001
	Gestation	0.31	<0.001
	Mother smoked	−0.18	<0.001
	Maternal education		
	Complete high school	0.06	0.08
	Post-high school	0.08	0.01
	Maternal height^a^	0.15	<0.001
	Maternal BMI	0.09	<0.001
PC2: adiposity	(Constant)		<0.001
	Male sex	−0.16	<0.001
	Gestation	0.07	0.009
	Multiparous	0.10	<0.001
	Maternal BMI	0.15	<0.001
PC3: limb lengths	(Constant)		<0.001
	Male sex	0.109	<0.001
	Gestation	0.165	<0.001
	Mother smoked	−0.07	0.02
	Paternal height	0.143	<0.001
	Paternal BMI	0.085	0.004

a Parental height and BMI are log values.

## DISCUSSION

The results show that various neonatal body measurements differ in their relationships to parental anthropometry. In the analyses of individual neonatal measurements, maternal height and BMI and paternal height related to offspring birthweight, both parents' height and BMI related to neck-rump length, and both parents' height to proximal limb segment lengths. Only paternal height and BMI related to distal limb segment length, maternal height and BMI to head circumference, and maternal BMI to adiposity.

Analyses of the PC scores highlighted similar patterns, and suggested associations of maternal height and BMI with offspring head and trunk skeletal size (PC1), maternal BMI with offspring adiposity (PC2), and paternal height and BMI with neonatal limb lengths (PC3). Previous studies have identified very similar PCs of neonatal anthropometric variation (Denham et al., 2001; Hindmarsh et al., 2002; Shields et al., 2006; Veena et al., 2009) and found similar relationships between the PCs and parental anthropometry, suggesting common underlying patterns of variation in neonatal size and shape despite genetic and socioeconomic differences between populations, and methodological differences between studies.

The relationship between birthweight and paternal height, but not BMI, and the stronger association of both maternal height and BMI with birthweight than paternal height are consistent with previous studies (Kramer, 1987; Morrison et al., 1991; To et al., 1998; Knight et al., 2005; Leary et al., 2006; Griffiths et al., 2007; Veena et al., 2009; Albouy-Llaty et al., 2011; Kuzawa and Eisenberg, 2012). However, other studies have not tested statistically the difference in the strength of maternal and paternal coefficients, and our results indicate that this difference is not significant.

Parental height and weight or BMI reflect both genetic factors as well as the parents' past (height and BMI) and current (BMI) environment, so associations between parental and offspring anthropometry reflect the transmission of heritable (genetic/epigenetic) influences on growth incorporating elements of the parents' developmental experience and current environment. The different relationships between maternal or paternal heights and various neonatal measurements have been interpreted as indicating stronger prenatal genetic regulation of skeletal growth than of adiposity (Godfrey et al., 1997; Knight et al., 2005; Leary et al., 2006; Shields et al., 2006; Veena et al., 2009; Sletner et al., 2013). However, our results suggest a more subtle interpretation based on linking differing parental “interests” in investment, obstetric constraints, maternal resource availability and contrasting parental influences on distinct components of early offspring skeletal growth.

The stronger association between maternal anthropometry and head circumference compared with paternal anthropometry, and the exclusive association between the “head and trunk skeletal size” PC and maternal anthropometry, might reflect processes that serve to prevent a mismatch between fetal size and maternal birth canal dimensions that could otherwise result in obstructed labor (Pembrey, 1996). Maternal height correlates positively with her pelvic dimensions and is an important predictor of obstructed labor (Connolly and McKenna, 2001; Kjærgaard et al., 2010; Benjamin et al., 2012). Furthermore, in the “head and trunk skeletal size” PC, trunk breadths feature relatively prominently along with head size. Given that shoulder dystocia is an important cause of obstructed labor that has been linked to humans' relatively broad shoulders (Trevathan and Rosenberg, 2000), this may also suggest maternal constraints on fetal head and trunk size to prevent cephalo-pelvic disproportion. Indeed, Veena et al. (2009) reported that in an Indian sample, maternal external pelvic dimensions were an independent predictor of neonatal skeletal head and trunk size, and that maternal height and BMI were much more strongly associated with their neonatal head and trunk PC score than those of the father, consistent with our results and interpretation. Maternal height may thus be associated with overall newborn size due to shared genotype and to prevent obstructed labor.

Maternal BMI indicates aspects of the fetal environment, since maternal BMI may have direct physiological influences on fetal growth through determining, for example, nutrient supply and hormone profiles (King, 2006; Jansson et al., 2008; Higgins et al., 2011). Thus maternal BMI may be associated with neonatal fatness as it reflects maternal resource availability. Increasing neonatal fatness where resources permit may allow the mother to opportunistically enhance early infant growth and survival, with which birthweight and fatness are associated (Karn and Penrose, 1951; Wilcox and Russell, 1983; Wiley, 1994; Kuzawa, 1998).

Paternal anthropometry may be more closely associated with limb size since this enables the father's (epi)genotype to maximize fetal growth without coming up against strong maternal constraints that act to prevent obstructed labor. Paternal interests may be served by enhancing linear (particularly limb) growth, since this avoids exacerbating obstetric risks while driving greater lean mass accretion. Greater birthweight and length are positively associated with greater adult height and lean mass (Sørensen et al., 1999; Pietiläinen et al., 2002; Eide et al., 2005; Adair, 2007; Wells et al., 2007), and greater height is associated with enhanced reproductive success in both males (Pawlowski et al., 2000; Nettle, 2002; Sear, 2006) and (non-Western) females (Martorell et al., 1981; Sear et al., 2004; Sear, 2006; Pollet and Nettle, 2008). By influencing early lean tissue growth the father may ultimately enhance his offspring's reproductive success.

As BMI reflects both fat and lean mass, and height is associated with lean mass, the pattern of association between neonatal limb dimensions and paternal anthropometry could reflect a link between paternal lean mass and skeletal size at birth. Previous studies report that paternal height is significantly associated with neonatal fat free mass (Catalano et al., 1995), bone mass (Godfrey et al., 2001), and arm circumference but not skinfolds or birthweight (Knight et al., 2005), suggesting a paternal size effect on neonatal lean mass. Lean mass in fetal life may also then track into adulthood, since associations between birthweight and adult lean mass, but not fat mass, have been documented (Singhal et al., 2003; Sachdev et al., 2005; Wells et al., 2007).

The extent to which relationships between parental and offspring anthropometry are genetic, epigenetic or phenotypic in origin is currently unclear. Epigenetics may play an important role in associations between paternal and offspring metabolism (Kaati et al., 2002; Pembrey, 2002; Lecomte et al., 2013; Wells, 2014), and could also link to prenatal growth. For example, Soubry et al. (2013) recently showed that paternal obesity was associated with hypomethylation of *IGF2*, an important regulator of prenatal growth. The *IGF2* gene is paternally expressed and maternally imprinted in the placenta, and expression during the first trimester is positively associated with offspring birthweight (Demetriou et al., 2014). Thus *IGF2* expression, particularly in early pregnancy, may play a role in early offspring growth, but a number of imprinted loci relating to fetal and neonatal size have been identified in humans that seem to have effects at different times during pregnancy (Hitchins and Moore, 2002; Apostolidou et al., 2007; Ishida et al., 2012; Kumar et al., 2012; Ishida and Moore, 2013; Demetriou et al., 2014). Furthermore the pattern of imprinting may relate to prepregnancy and *in utero* environment, as well as offspring sex (Tobi et al., 2009). How maternal and paternal genes are expressed in the growing fetus, and the extent to which their expression is mediated by environmental factors, is a complex area which were are only now beginning to understand.

Previous studies have rarely tested statistically for differences between sons and daughters in the relationship between neonatal and parental phenotype. Though there is suggestive evidence that parental phenotype and prenatal environment might affect the sexes differently (Pembrey et al., 2005; Anderson et al., 2006; Thone-Reineke et al., 2006; Chen et al., 2012; Aiken and Ozanne, 2013), reported patterns are rarely tested statistically. Such differences might be predicted theoretically since males are thought to place a greater demand on maternal physiology (Stinson, 1985) due to their faster growth and larger size (Catalano et al., 1995; Hindmarsh et al., 2002; Melamed et al., 2013), and to be more sensitive to early growth disturbance (Stini, 1969; Stinson, 1985; Kuh et al., 1991; Wamani et al., 2007; Ashizawa et al., 2008; Decaro et al., 2010). However, we found no evidence of such a contrast in this study. As the study sample represents a western, relatively wealthy population, whether the same result would be found in more stressful environment where maternal energetics are more marginal remains to be tested.

Parental anthropometry explained a relatively low proportion of variance in neonatal measurements (<7%), indicating the importance of various environmental and genetic factors on both parental and neonatal phenotype. Documented associations between parental and offspring height are stronger in adulthood than at birth, with heritability estimates of around 80% in adulthood for relatively wealthy populations (Silventoinen et al., 2003). Heritability estimates of various measurements including head circumference, height and weight also increase from approximately 6 months of age compared with at birth when they are typically 25–30% (Levine et al., 1987; Demerath et al., 2007; Johnson et al., 2011; Silventoinen et al., 2011; Mook-Kanamori et al., 2012). Thus fetal growth may be generally more sensitive to the environment than postnatal growth, accounting for closer relationships between parental and offspring anthropometry in adulthood. This environmental sensitivity prior to birth may aid in preventing a mismatch between offspring genetic growth potential and maternal body size, which is the outcome of both genetics and past environment, and could raise the risk of obstructed labor (Wells, In press). Various studies of humans and other mammals indicate that maternal size acts to constrain fetal size (Walton and Hammond, 1938; Morton, 1955; Brooks et al., 1995; Wells et al., 2013), presumably to prevent such a mismatch.

The strengths of this study include the large sample size and range of anthropometric and other variables. Many previous studies derive leg length by subtracting crown-rump from crown-heel length, meaning these measurements are not independent and include head size in the total and trunk length measurements. In our dataset, trunk length (neck-rump length), head size and limb lengths were measured independently, permitting their individual associations with parental measurements and their contributions to neonatal anthropometric variation to be more readily separable and interpretable (e.g., Shields et al., 2006; Veena et al., 2009).

Paternal height and the weights of both parents were self reported, so subject to bias (Gorber et al., 2007; McAdams et al., 2007). However, BMI based on self-reported measurements may be sufficiently accurate for epidemiological studies (McAdams et al., 2007). Our analyses also did not include several other factors that have been previously shown to relate to neonatal anthropometry, including maternal pregnancy weight gain (Kramer, 1987; Catalano et al., 1995; Goldenberg et al., 1997; Frederick et al., 2008; Roland et al., 2012; Tikellis et al., 2012), maternal and paternal birthweight (Kramer, 1987; Little, 1987; Emanuel et al., 1992; Magnus et al., 2001), maternal micronutrient status (Kramer, 1987; Mathews et al., 1999; Leffelaar et al., 2010), placental weight (Kramer, 1987; Roland et al., 2012; Tikellis et al., 2012) and maternal glucose metabolism before or during pregnancy (Catalano et al., 2003; HAPO Study Cooperative Research Group, 2009; Catalano et al., 2012; Roland et al., 2012).

Non-paternity may have attenuated associations between paternal and neonatal phenotype. Paternity was not genetically tested in this dataset. Estimated rates of nonpaternity in western populations vary from <1% to >30% (Bellis et al., 2005; Anderson, 2006; Voracek et al., 2008). However, the higher estimates derive from studies where participants had already expressed doubt regarding paternity, biasing the samples (Bellis et al., 2005; Anderson, 2006; Voracek et al., 2008). Recent estimates suggest average nonpaternity rates of 1–3% in the general population (Bellis et al., 2005; Anderson, 2006; Voracek et al., 2008; Wolf et al., 2012; Larmuseau et al., 2013). Thus given the generally low rates of nonpaternity in western populations, this likely had a relatively small influence on our results.

In conclusion, our results suggest that variation in neonatal body measurements may be represented by “head and trunk skeletal size,” “adiposity,” and “limb length” components, and that different individual measurements or components vary in their associations with parental anthropometry. Paternal body size was particularly associated with limb lengths, while maternal height and BMI were more strongly associated with adiposity and birthweight. We suggest that this may reflect the need to tailor fetal head and trunk size to maternal pelvic dimensions in order to reduce the risk of obstructed labor. Paternal factors may increase maternal physiological investment in the fetus without exacerbating obstetric risks by driving greater limb lengths and lean tissue. While the relationship between neonatal skinfolds and maternal BMI likely reflects an environmental effect on fetal growth while limb and trunk size are more strongly genetically determined, the extent to which parental phenotype mediates head, trunk and limb sizes is unclear. The implications of this study are that neonatal anthropometric phenotype is represented by similar key components across multiple populations, regardless of ethnicity and SES, and that environmental factors, obstetric constraints and parental conflicts of interest may lead to different associations between maternal or paternal body size and distinct components of neonatal phenotype.
