# Supplementary material for: Relationships of maternal and paternal anthropometry with neonatal body size, proportions and adiposity in an Australian cohort
Source: Am J Phys Anthropol. 2014 Dec 13;156(4):625–36. doi: 10.1002/ajpa.22680 (PMC4404025; doi:10.1002/ajpa.22680)
Supplement: Supplementary file 1 — Supplementary Information [file ajpa0156-0625-sd1.doc]

**SUPPLEMENTARY INFORMATION**

**Supplementary Table 1: Final regression models of neonatal anthropometry on parental anthropometry: regression coefficients and significance of potential confounders included in the model**

| **Dependent** | **Body measurement ^a^** | | | **Male sex** | | **Gestation** | | **Mother smoked** | | **Maternal education ^b^** | | | | **Previous birth** | | **Maternal age** | | **Adjusted R square^c^** |
| --- | --- | --- | --- | --- | --- | --- | --- | --- | --- | --- | --- | --- | --- | --- | --- | --- | --- | --- |
|  |  |  |  |  |  |  |  |  |  | **Complete high** | | **Post high** | |  |  |  |  |  |
|  | **Variable** | **β** | **p** | **β** | **p** | **β** | **p** | **β** | **p** | **β** | **p** | **β** | **p** | **β** | **p** | **β** | **p** |  |
| Birth weight |  |  |  | 0.13 | <0.001 | 0.33 | <0.001 | -0.20 | <0.001 | 0.08 | 0.02 | 0.08 | 0.03 | 0.07 | 0.01 | 0.06 | 0.03 | 0.23 |
| Neck-rump length |  |  |  | 1.26 | 0.05 | 0.22 | <0.001 | -0.10 | <0.001 | 0.08 | 0.04 | 0.11 | 0.006 | 0.09 | 0.004 |  |  | 0.12 |
| Head circumference |  |  |  | 0.23 | <0.001 | 0.33 | <0.001 | -0.10 | <0.001 | 0.10 | 0.004 | 0.09 | 0.01 |  |  |  |  | 0.21 |
| Sum of 4 skinfolds |  |  |  | -0.12 | <0.001 | 0.15 | <0.001 |  |  | 0.08 | 0.03 | 0.08 | 0.05 | 0.11 | 0.001 |  |  | 0.06 |
| Upper arm length |  |  |  | 0.09 | 0.003 | 0.17 | <0.001 | -0.10 | 0.002 | 0.03 | 0.4 | 0.07 | 0.09 |  |  |  |  | 0.06 |
| Lower arm length |  |  |  | 0.08 | 0.007 | 0.13 | <0.001 |  |  |  |  |  |  |  |  |  |  | 0.04 |
| Thigh length |  |  |  | 0.06 | 0.04 | 0.20 | <0.001 | -0.11 | <0.001 | *0.07* | *0.08* | *0.07* | *0.06* |  |  | 0.06 | 0.05 | 0.09 |
| Lower leg length |  |  |  | *0.071* | *0.02* | 0.14 | <0.001 |  |  | 0.05 | 0.22 | 0.08 | 0.05 |  |  |  |  | 0.04 |
| Relative upper limb length | Neck-rump length | 0.20 | <0.001 | 0.081 | 0.007 | 0.11 | <0.001 |  |  |  |  |  |  |  |  |  |  | 0.08 |
| Relative lower limb length | Neck-rump length | 0.22 | <0.001 | 0.06 | 0.04 | 0.13 | <0.001 | -0.07 | 0.02 |  |  |  |  |  |  |  |  | 0.10 |
| ‘Brachial index’ | Upper arm length | 0.72 | <0.001 |  |  |  |  | 0.04 | 0.08 |  |  |  |  |  |  |  |  | 0.52 |
| ‘Crural index’ | Thigh length | 0.71 | <0.001 |  |  |  |  |  |  |  |  |  |  |  |  |  |  | 0.50 |

^a^ Only included in models for relative limb length indices

**^b^** Reference category = incomplete high school education or less. ‘Complete high’ = completed high school; ‘Post high’ = post high school education.

**^c^** Adjusted R^2^ for all adjustors in the model combined

*Italics* indicate variables where 0.05<p<0.1.
