# Supplementary material for: Relationships of maternal and paternal anthropometry with neonatal body size, proportions and adiposity in an Australian cohort
Source: Am J Phys Anthropol. 2014 Dec 13;156(4):625–36. doi: 10.1002/ajpa.22680 (PMC4404025; doi:10.1002/ajpa.22680)
Supplement: Supplementary file 3 — Supplementary Information [file ajpa0156-0625-sd3.doc]

**TABLES**

**Table 1: Neonatal characteristics of the study sample**

| **Characteristic** | **Female** | | | **Male** | | | | **Combined** | | | |
| --- | --- | --- | --- | --- | --- | --- | --- | --- | --- | --- | --- |
|  | **Mean** | | **SD** | | **Mean** | | **SD** | | **Mean** | **SD** | |
| Birth weight (g) | 3399 | | 450 | | 3521 | | 430 | | 3463 | 440 | |
| Head circumference (mm) | 348 | | 12 | | 355 | | 12 | | 352 | 12 | |
| Biparietal diameter (mm) | 94 | | 3.5 | | 95 | | 3.6 | | 95 | 3.6 | |
| Face diameter (mm) | 86 | | 4.1 | | 87 | | 4.3 | | 86 | 4.3 | |
| Neck-rump length (mm) | 227 | | 15 | | 229 | | 14 | | 228 | 15 | |
| Shoulders width (mm) | 157 | | 9.8 | | 159 | | 11 | | 158 | 10 | |
| Hips width (mm) | 133 | | 10 | | 134 | | 11 | | 133 | 11 | |
| Upper arm length (mm) | 83 | | 6.6 | | 85 | | 6.9 | | 84 | 6.8 | |
| Upper arm circumference (mm) | 109 | | 9.2 | | 110 | | 9.0 | | 110 | 9.1 | |
| Lower arm length (mm) | 60 | | 8.2 | | 62 | | 7.9 | | 61 | 8.1 | |
| Lower arm circumference (mm) | 100 | | 7.7 | | 101 | | 7.2 | | 100 | 7.4 | |
| Chest circumference (mm) | 333 | | 17 | | 335 | | 17 | | 334 | 17 | |
| Abdomen circumference (mm) | 289 | | 20 | | 288 | | 17 | | 288 | 19 | |
| Thigh length (mm) | 89 | | 6.8 | | 90 | | 6.7 | | 90 | 6.8 | |
| Thigh circumference (mm) | 155 | | 14 | | 154 | | 13 | | 155 | 14 | |
| Lower leg length (mm) | 68 | | 7.9 | | 70 | | 8.1 | | 69 | 8.0 | |
| Lower leg circumference (mm) | 113 | | 8.6 | | 113 | | 8.3 | | 113 | 8.4 | |
| Skinfold subscapular (mm) | 55 | | 10 | | 52 | | 10 | | 54 | 10 | |
| Skinfold abdominal (mm) | 35 | | 6.0 | | 35 | | 6.3 | | 35 | 6 | |
| Skinfold triceps (mm) | 50 | | 9.1 | | 49 | | 8.8 | | 49 | 9.0 | |
| Skinfold anterior thigh (mm) | 67 | | 14 | | 63 | | 14 | | 65 | 14 | |
| Gestational age (wks) | 40 | | 1.2 | | 40 | | 1.3 | | 40 | 1.2 | |
|  |  |  | |  | |  | |  | | |  |

**Table 2: Parental characteristics of the study sample**

| ***Continuous variables*** | | **Offspring sex** | | | | | |
| --- | --- | --- | --- | --- | --- | --- | --- |
|  |  | **Female (n=492)** | | **Male (n=549)** | | **Combined (n=1041)** | |
|  |  | **Mean** | **SD** | **Mean** | **SD** | **Mean** | **SD** |
| **Maternal height (cm)** | | 163 | 6.3 | 163 | 6.2 | 163 | 6.2 |
| **Maternal BMI (kg/m^2^)** | | 21.8 | 3.7 | 22.1 | 4.2 | 22.0 | 4.0 |
| **Paternal height (cm)** | | 177 | 7.9 | 176 | 7.9 | 176 | 7.9 |
| **Paternal BMI (kg/m^2^)** | | 23.5 | 3.4 | 23.6 | 3.7 | 23.6 | 3.5 |
| **Maternal age (years)** | |  |  |  |  |  |  |
| ***Categorical variables*** | | **n (%)** | | **n (%)** | | **n (%)** | |
| **Maternal education** | **Incomplete high school** | 89 (18) | | 91 (17) | | 180 (17) | |
|  | **Complete high school** | 320 (65) | | 359 (65) | | 679 (65) | |
|  | **Post-high school** | 83 (17) | | 99 (18) | | 182 (18) | |
| **Maternal smoking** | **No** | 300 (61) | | 353 (64) | | 653 (63) | |
|  | **Yes** | 192 (39) | | 196 (36) | | 388 (37) | |
| **Parity** | **0** | 194 (39) | | 231 (42) | | 425 (41) | |
|  | **1+** | 298 (61) | | 318 (58) | | 616 (59) | |

**Table 3: Final regression models of neonatal anthropometry on parental anthropometry, adjusting for potential confounders**

| **Measurement** | **Maternal height** | | **Maternal BMI** | | **Paternal height** | | **Paternal BMI** | | **Adjusted R^2^** |
| --- | --- | --- | --- | --- | --- | --- | --- | --- | --- |
|  | **β** | **p** | **β** | **p** | **β** | **p** | **β** | **p** |  |
| Birth weight | 0.17 | <0.001 | 0.16 | <0.001 | 0.08 | 0.003 |  |  | 0.06 |
| Neck-rump length | 0.12 | <0.001 | 0.09 | 0.002 | 0.11 | <0.001 | 0.12 | 0.008 | 0.05 |
| Head circumference | 0.10 | <0.001 | 0.14 | <0.001 |  |  |  |  | 0.03 |
| Sum of 4 skinfolds |  |  | 0.14 | <0.001 |  |  |  |  | 0.02 |
| Upper arm length | 0.08 | 0.007 |  |  | 0.10 | 0.001 |  |  | 0.02 |
| Lower arm length |  |  |  |  | 0.12 | <0.001 | 0.09 | 0.006 | 0.02 |
| Thigh length | 0.10 | 0.001 | 0.08 | 0.008 | 0.08 | 0.008 |  |  | 0.02 |
| Lower leg length |  |  |  |  | 0.12 | <0.001 | 0.09 | 0.003 | 0.02 |
| Relative upper limb length |  |  |  |  | 0.10 | 0.002 |  |  | 0.01 |
| Relative lower limb length |  |  |  |  | 0.09 | 0.004 |  |  | 0.01 |
| ‘Brachial index’ |  |  |  |  |  |  |  |  | 0.00 |
| ‘Crural index’ |  |  |  |  |  |  |  |  | 0.00 |

All variables log transformed.

**Table 4. Variable loadings for the first three principal components from principal components analysis of neonatal anthropometry**

| **Measurement** | **Unrotated component** | | | **Varimax rotated component** | | |
| --- | --- | --- | --- | --- | --- | --- |
|  | **1** | **2** | **3** | **1** | **2** | **3** |
| Birth weight | **0.93** | -0.04 | 0.11 | **0.69** | 0.52 | 0.38 |
| Head circumference | **0.74** | -0.05 | 0.39 | **0.76** | 0.20 | 0.29 |
| Biparietal width | **0.60** | -0.24 | 0.54 | **0.84** | 0.05 | 0.07 |
| Face width | 0.49 | -0.52 | 0.50 | **0.83** | 0.08 | -0.23 |
| Neck-rump length | 0.57 | -0.03 | 0.26 | 0.55 | 0.18 | 0.24 |
| Shoulder width | **0.71** | -0.24 | 0.19 | **0.67** | 0.37 | 0.11 |
| Hip width | **0.63** | -0.36 | 0.18 | **0.65** | 0.37 | -0.04 |
| Upper arm length | 0.57 | **0.63** | 0.07 | 0.19 | 0.14 | **0.82** |
| MUAC | **0.86** | 0.00 | -0.09 | 0.49 | **0.60** | 0.37 |
| Lower arm length | 0.42 | **0.78** | -0.02 | -0.01 | 0.07 | **0.89** |
| Lower arm circumference | **0.88** | -0.03 | -0.09 | 0.51 | **0.62** | 0.36 |
| Chest circumference | **0.83** | 0.11 | 0.06 | 0.54 | 0.45 | 0.47 |
| Abdomen circumference | **0.82** | 0.12 | -0.06 | 0.44 | 0.52 | 0.46 |
| Thigh length | 0.57 | **0.66** | 0.11 | 0.21 | 0.10 | **0.84** |
| Thigh circumference | **0.82** | -0.12 | -0.10 | 0.51 | **0.62** | 0.24 |
| Lower leg length | 0.41 | **0.79** | 0.02 | 0.00 | 0.03 | **0.89** |
| Lower leg circumference | **0.88** | -0.05 | -0.10 | 0.51 | **0.64** | 0.33 |
| Subscapular skinfold | **0.65** | -0.24 | -0.52 | 0.15 | **0.85** | 0.05 |
| Abdominal skinfold | **0.61** | 0.11 | -0.47 | 0.03 | **0.69** | 0.34 |
| Triceps skinfold | 0.53 | -0.35 | -0.46 | 0.15 | **0.76** | -0.10 |
| Anterior thigh skinfold | **0.65** | -0.38 | -0.41 | 0.27 | **0.82** | -0.08 |
|  |  |  |  |  |  |  |
| *Variance explained (%)* | *47.8* | *14.1* | *8.3* | *25.3* | *24.3* | *20.6* |

**Bold** indicates loadings ≥|0.6|.

**Table 5: Regression analysis of principal component (PC) scores from neonatal anthropometry on parental anthropometry and potential confounding variables**

|  | **Standardized coefficient (β)** | **p** |
| --- | --- | --- |
| **PC1: head & trunk skeletal size** | | |
| (Constant) |  | <0.001 |
| Male sex | 0.21 | <0.001 |
| Gestation | 0.31 | <0.001 |
| Mother smoked | -0.18 | <0.001 |
| Maternal education: complete high school | 0.06 | 0.08 |
| post high school | 0.08 | 0.01 |
| Maternal height ^a^ | 0.15 | <0.001 |
| Maternal BMI | 0.09 | <0.001 |
| **PC2: adiposity** |  |  |
| (Constant) |  | <0.001 |
| Male sex | -0.16 | <0.001 |
| Gestation | 0.07 | 0.009 |
| Multiparous | 0.10 | <0.001 |
| Maternal BMI | 0.15 | <0.001 |
| **PC3: limb lengths** |  |  |
| (Constant) |  | <0.001 |
| Male sex | 0.109 | <0.001 |
| Gestation | 0.165 | <0.001 |
| Mother smoked | -0.07 | 0.02 |
| Paternal height | 0.143 | <0.001 |
| Paternal BMI | 0.085 | 0.004 |

^a^ Parental height and BMI are log values.
