# Supplementary material for: Relationships of maternal and paternal anthropometry with neonatal body size, proportions and adiposity in an Australian cohort
Source: Am J Phys Anthropol. 2014 Dec 13;156(4):625–36. doi: 10.1002/ajpa.22680 (PMC4404025; doi:10.1002/ajpa.22680)
Supplement: Supplementary file 4 — Supplementary Information [file ajpa0156-0625-sd4.doc]

**Title: Relationships of maternal and paternal anthropometry with neonatal body size, proportions and adiposity in an Australian cohort**

**Authors:** Emma Pomeroy ^a,b^

Jonathan C.K. Wells ^b,c^

Tim J. Cole ^d^

Michael O’Callaghan ^e^

Jay T. Stock ^b^

**Affiliations**

^a^ Newnham College, University of Cambridge, UK

^b^ Division of Biological Anthropology, Department of Archaeology and Anthropology, University of Cambridge, UK

^c^ Childhood Nutrition Research Centre, UCL Institute of Child Health, UK

^d^ Population Policy and Practice Programme, UCL Institute of Child Health, UK

^e^ School of Medicine, Mater Clinical School, University of Queensland, Brisbane, Australia

**Number of:** 14 text pages (+ 14 bibliography), 2 figures, 3 tables

**Abbreviated title:** Maternal, paternal and neonatal anthropometry

**Key words:** neonatal anthropometry; birthweight; limb length; parental height; parental BMI

Corresponding author: Dr Emma Pomeroy

Newnham College

Cambridge CB3 9DF

UK

Tel: +44 7815 189784

Email: emma.pomeroy@cantab.net

**Grant sponsorship:** The relevant phases of the Mater-University of Queensland Study of Pregnancy were funded by the National Health and Medical Research Council (Australia). EP is funded by the Henry Sidgwick Research Fellowship from Newnham College, Cambridge, UK. TJC is funded by Medical Research Council grant MR/J004839/1.

**ABSTRACT**

The patterns of association between maternal or paternal and neonatal phenotype may offer insight into how neonatal characteristics are shaped by evolutionary processes, such as conflicting parental interests in fetal investment and obstetric constraints. Paternal interests are theoretically served by maximizing fetal growth, and maternal interests by managing investment in current and future offspring, but whether paternal and maternal influences act on different components of overall size is unknown. We tested whether parents’ pre-pregnancy height and BMI were related to neonatal anthropometry (birthweight, head circumference, absolute and proportional limb segment and trunk lengths, subcutaneous fat) among 1041 Australian neonates using stepwise linear regression. Maternal and paternal height and maternal BMI were associated with birthweight. Paternal height related to offspring forearm and lower leg lengths, maternal height and BMI to neonatal head circumference, and maternal BMI to offspring adiposity. Principal components analysis identified three components of variability reflecting neonatal ‘head and trunk skeletal size’, ‘adiposity’, and ‘limb lengths’. Regression analyses of the component scores supported the associations of head and trunk size or adiposity with maternal anthropometry, and limb lengths with paternal anthropometry. Our results suggest that while neonatal fatness reflects environmental conditions (maternal physiology), head circumference and limb and trunk lengths show differing associations with parental anthropometry. These patterns may reflect genetics, parental imprinting and environmental influences in a manner consistent with parental conflicts of interest. Paternal height may relate to neonatal limb length as a means of increasing fetal growth without exacerbating the risk of obstetric complications.

Fetal growth and development have important implications across the life-course, influencing the risk of birth complications (Koyanagi et al., 2013), neonatal morbidity and mortality (Karn and Penrose, 1951; McIntire et al., 1999), the schedule and trajectory of postnatal growth (Smith et al., 1976; Mei et al., 2004), reproductive function (Lummaa, 2003) and adult disease risk (Hales and Barker, 1992; Barker, 1998). Given the extensive implications of early growth and development, we might expect the prenatal period to be an important stage at which parental genetic, epigenetic or phenotypic factors may influence offspring phenotype. Understanding these influences on fetal growth may offer insights into the evolutionary processes affecting early development.

The genotype and phenotype of both parents are associated with fetal and neonatal phenotype (Lindsay et al., 2000; Hyppönen et al., 2003; Anderson et al., 2006; Carone et al., 2010; Ng et al., 2010; Myklestad et al., 2012; Hillman et al., 2013; Tyrrell et al., 2013; Wells et al., 2013; Wei et al., 2014). From an evolutionary perspective, parents may have conflicting ‘interests’ in early offspring growth (Haig and Westoby, 1989; Moore and Haig, 1991). As the mother provides all the prenatal physiological investment, her lifetime reproductive success will be maximized by balancing investment in current and future offspring, since she will be equally related to each of them. In contrast, the father’s interests are best served by maximizing maternal investment in the current offspring, since her prior and/or subsequent offspring may not be his. Parental genes may therefore be involved in a ‘tug-of-war’ over maternal resources, with paternal genes promoting and maternal genes constraining fetal growth (Haig and Westoby, 1989; Moore and Haig, 1991).

Studies of humans and using animal models suggest that parental genes influence different aspects of placental size and physiology to promote (paternal) or restrict (maternal) fetal growth in a manner consistent with parental conflict theory (Willison, 1991; Allen et al., 2002a; Allen et al., 2002b; Hitchins and Moore, 2002; Apostolidou et al., 2007; Demetriou et al., 2014). For example, expression levels of paternally-expressed genes (e.g. *IGF2*) are positively associated and those of maternally-expressed genes (e.g. *PHLDA2*) negatively associated with birthweight (reviewed in Ishida and Moore, 2013).

Other constraints are also likely to influence fetal development, such as maternal obstetric dimensions (reviewed in Rosenberg and Trevathan, 2002; Wells et al., 2012; Pomeroy et al., In press). Environmentally-responsive aspects of maternal phenotype including height and pelvic geometry (Liselele et al., 2000; Kjærgaard et al., 2010; Benjamin et al., 2012), and neonatal characteristics including head and shoulder dimensions (Trevathan and Rosenberg, 2000; Rosenberg and Trevathan, 2002), likely contribute to the risk of obstructed labour resulting from a mismatch between fetal size and maternal pelvic dimensions. Associations between grandmaternal malnutrition and newborn size, and secular increases in birthweight, suggests that fetal development is ‘tailored’ to current maternal pelvic dimensions to avoid such obstetric complications (Pembrey 1996). While fathers lose potential reproductive success if the offspring and mother die through obstructed labour, the penalty in lifetime reproductive success is much greater for the mother if she dies in childbirth, creating further tension between maternal and paternal interests in fetal growth.

It is unknown whether the outcome of this parental ‘tug of war’ may also lead to differing associations between parental phenotype and distinct components of fetal growth, but detailed analyses of neonatal phenotype (limb, trunk and head size, adiposity) may offer insight into this question. We therefore examined associations of maternal and paternal anthropometry (height, BMI) with offspring characteristics including birthweight, head circumference, absolute and proportional limb segment and trunk lengths, and skinfolds. We hypothesized that maternal and paternal anthropometry would show differing associations with different components of neonatal phenotype.

**MATERIALS AND METHODS**

We analyzed data on neonatal and parental anthropometry from the Mater-University of Queensland Study of Pregnancy (MUSP) dataset (Najman et al., 2005). The study was approved by ethics committees from the Mater Hospitals and the University of Queensland, and maternal oral informed consent was obtained (in keeping with standards at the time of this phase of the study in the early 1980s). The study recruited 7223 mother-infant pairs in Brisbane, Australia, although detailed anthropometry that included limb segment lengths was only collected in the subset of neonates (n=1271 live singleton births, 668 males) born between 1982-3, on which our analysis focuses. For the present analyses, the dataset was further limited to individuals with complete anthropometry and explanatory variables, and two infants with anomalous measurements or multiple congenital anomalies were also omitted, leaving a total sample of 1041 neonates (Fig 1).

*** INSERT FIGURE 1 HERE ***

McGrath et al. (2005) reported that there were no significant differences in birthweight or sex ratio between the full sample and the sample for which detailed anthropometry were recorded, except a small difference in gestational age that was statistically, but unlikely to be biologically, significant (0.1 weeks longer among included neonates; p <0.01). Maternally-reported ethnicity of the parents in the sample was overwhelmingly ‘White’ (91% of 1216 mothers and 93% of 1167 fathers on whom data were available, remaining parents split approximately equally between ‘Asian’ and ‘Aboriginal/Islander’).

All babies were measured by a trained research nurse (Keeping, 1981; McGrath et al., 2005) within 24 hours of birth. No data on inter-rater reliability are available. The neonatal measurements in this analysis were: birthweight; head, abdominal, upper arm, lower arm, thigh and lower leg circumferences; face, biparietal, shoulder and hip breadths; neck-rump, upper arm, forearm, thigh, and lower leg lengths; and subscapular, triceps, abdominal and anterior thigh skinfolds. Data were confirmed graphically to follow a normal distribution.

Maternal height (to nearest cm) was measured at the first prenatal clinic visit, while paternal height (to nearest cm) and weight, and maternal pre-pregnancy weight (to nearest kg), were self-reported. They were used to calculate parental BMI, and natural logarithms of parental height and BMI formed the primary explanatory variables. Sex and gestational age (e.g. Catalano et al., 1995; Hindmarsh et al., 2002; Knight et al., 2005; Shields et al., 2006) and several potential confounding variables (maternal smoking, education, parity: Kramer et al., 2000; Raum et al., 2001; Voigt et al., 2004; Harvey et al., 2007; Elshibly and Schmalisch, 2009; Jansen et al., 2009; van den Berg et al., 2013) were included in the analyses based on associations reported in the literature. Potential confounders were recorded at the first clinic visit or extracted from medical records. Parity was coded as 0 vs. 1 or more. Maternal education was coded into dummy variables for 3 categories: incomplete- (reference), complete- and post- high school. Maternal smoking in the last trimester was coded as yes or no, and maternal age at birth in years was also recorded. Data were available on family income but were omitted from analyses as they were not significant in the regression models.

Multiple regression was used to analyze the relationship between neonatal body measurements (as dependent variables) and parental height and BMI, adjusted for the potential confounding variables specified in the Results. Dependent variables were natural logarithms of head circumference, neck-rump length, upper arm length, forearm length, thigh length, lower leg length, birthweight, and sum of 4 skinfolds (subscapular, triceps, abdominal and anterior thigh), as well as the following limb proportions calculated from the log transformed data: relative upper (upper arm length + forearm length, adjusting for neck-rump length in the regression model) and lower limb lengths (thigh length + lower leg length, adjusting for neck-rump length); and intralimb indices: brachial (forearm length adjusting for upper arm length in the regression model) and crural index (lower leg length adjusting for thigh length). Neonatal measurements were selected to represent diverse aspects of neonatal phenotype, including fatness and head, trunk and limb dimensions. The proportions of limb to trunk lengths were calculated to further highlight any differing relationships between the different body segments and parental anthropometry that may exist. The relative lengths of the distal (forearm or lower leg) to proximal (upper arm or thigh) limb bones were calculated since distal limb segment lengths may be particularly sensitive to environmental growth disturbance (Meadows Jantz and Jantz, 1999; Lampl et al., 2003; Bailey et al., 2007; Pomeroy et al., 2012; 2013), but the relationship between neonatal intralimb proportions and parental anthropometry is unknown.

Male sex, gestational age (weeks) and potential confounders were entered in the first round of the regression model where p<0.1. Parental heights and BMIs were entered in the second round using a stepwise procedure, with p<0.01 (rather than 0.05 due to the number of analyses performed. Interaction terms between offspring sex and parental anthropometry variables were also tested for. Where the equivalent anthropometry of both parents was significant in the model (e.g. both parents’ heights), we ran an otherwise identical regression model where parental heights were replaced with log geometric mean and log ratio of the two parents’ heights. The significance of the log ratio term indicates the significance of the difference in maternal and paternal regression coefficients, and p<0.01 was considered significant due to multiple analyses.

To further explore the relationships between maternal, paternal and neonatal anthropometry, all available neonatal measurements were submitted to principal components analysis (PCA) with varimax rotation to maximize the distinction between components and facilitate interpretation (Kaiser, 1958). PCA reduces the variables to a smaller set of variables, or principal components (PCs), which are linear combinations of the original variables that explain the majority of the variance in those variables (Dunteman, 1989). Each of the component scores for the first three PCs was analyzed with multiple regression as described for the original data. PCA was performed on pooled sex data since initial analyses (not shown) demonstrated little sex difference. Analyses were performed using SPSS version 21.0 for Windows.

**RESULTS**

The characteristics of the study sample are summarized in Tables 1-2. Five hundred and forty nine neonates were male (53%), and mean birthweights of males and females were 3.52kg and 3.40kg, very close to the medians (3.38kg and 3.26kg for males and females respectively) from recent international standards (Villar et al., 2014). Seven of the 1043 babies (0.7%) were of low birthweight (i.e. <2.5kg). Mean height and BMI were 163 cm and 22 kg/m^2^ for the mothers and 176 cm and 23.6 kg/m^2^ for the fathers. Forty-one percent were first births, 37% of mothers smoked, and mean maternal age at the child’s birth was 25.8 years.

*** INSERT TABLES 1-2 HERE ***

The adjusted R^2^ values for the final regression models (Fig. 1, Table 3) indicated that adjusting for potential confounders (see Supplementary Table 2 for details of confounders in each model), parental anthropometry explained a small proportion of variance in neonatal anthropometry. Parental measurements explained the greatest amount of variation in birthweight (6%) and neck-rump length (5%), but less variance in head circumference (3%), summed skinfolds (2%), limb segment lengths (2%), and limb proportion indices (0-1%). Birthweight was significantly associated with maternal height and BMI and paternal height (Fig. 2, Table 3). Associations were twice as strong for maternal vs. paternal height, but not statistically different (p=0.03). Neck-rump length related similarly to both parents’ heights and BMIs, with no significant differences between parental height or BMI coefficients (p>0.1). Head circumference related to maternal height and BMI only, and the sum of four skinfolds was only associated with maternal BMI. Proximal limb segment lengths (upper arm, thigh) related equally strongly to paternal and maternal height (p>0.1 in all tests for differences in parental height coefficients). In addition, maternal BMI was significantly associated with neonatal thigh length. Distal limb segments (lower arm, lower leg) were associated only with paternal anthropometry (both height and BMI). Limb: trunk length indices were associated with paternal height only, and intralimb indices did not relate to parental anthropometry. Offspring sex by parental anthropometry interactions were excluded from the models as they were not significant.

*** INSERT FIGURES 1-2 AND TABLE 3 HERE ***

The PCA analysis showed the same general patterns. Three PCs were derived using varimax rotation (Table 4). PC1 represented ‘head and trunk skeletal size’, as head circumference and breadths were most strongly loaded, followed by shoulder and hip widths and birthweight. PC2 represented ‘adiposity’, as skinfold thicknesses had the highest loadings, followed by limb circumferences (which reflect both adipose and lean tissue). PC3 represented ‘limb lengths’. Multiple regression analysis indicated significant positive associations between PC1 (‘head and trunk skeletal size’) and male offspring sex, gestational age, and maternal education, height and BMI, and a negative relationship with maternal smoking (Table 5). PC2 scores (‘adiposity’) related positively to gestational age, parity and maternal BMI and were lower among sons. PC3 (‘limb lengths’) was positively associated with male offspring sex, gestational age, and paternal height and BMI (Table 3), and negatively with maternal smoking.

*** INSERT TABLES 4-5 HERE ***

**DISCUSSION**

The results show that various neonatal body measurements differ in their relationships to parental anthropometry. In the analyses of individual neonatal measurements, maternal height and BMI and paternal height related to offspring birthweight, both parents’ height and BMI related to neck-rump length, and both parents’ height to proximal limb segment lengths, Only paternal height and BMI related to distal limb segment length, maternal height and BMI to head circumference, and maternal BMI to adiposity.

Analyses of the PC scores highlighted similar patterns, and suggested associations of maternal height and BMI with offspring head and trunk skeletal size (PC1), of maternal BMI with offspring adiposity (PC2), and paternal height and BMI with neonatal limb lengths (PC3). Previous studies have identified very similar principal components of neonatal anthropometric variation (Denham et al., 2001; Hindmarsh et al., 2002; Shields et al., 2006; Veena et al., 2009) and found similar relationships between the PCs and parental anthropometry, suggesting common underlying patterns of variation in neonatal size and shape despite genetic and socioeconomic differences between populations, and methodological differences between studies.

The relationship between birthweight and paternal height, but not BMI, and the stronger association of both maternal height and BMI with birthweight than paternal height are consistent with previous studies (Kramer, 1987; Morrison et al., 1991; To et al., 1998; Knight et al., 2005; Leary et al., 2006; Griffiths et al., 2007; Veena et al., 2009; Albouy-Llaty et al., 2011; Kuzawa and Eisenberg, 2012). However, other studies have not tested statistically the difference in strength of maternal and paternal coefficients, and our results indicate that this difference is not significant.

Parental height and weight or BMI reflect both genetic factors as well as the parents’ past (height and BMI) and current (BMI) environment, so associations between parental and offspring anthropometry reflect the transmission of heritable (genetic/epigenetic) influences on growth incorporating elements of the parents’ developmental experience and current environment. The different relationships between maternal or paternal heights and various neonatal measurements have been interpreted as indicating stronger prenatal genetic regulation of skeletal growth than of adiposity (Godfrey et al., 1997; Knight et al., 2005; Leary et al., 2006; Shields et al., 2006; Veena et al., 2009; Sletner et al., 2013). However, our results suggest a more subtle interpretation based on linking differing parental ‘interests’ in investment, obstetric constraints, maternal resource availability and contrasting parental influences on distinct components of early offspring skeletal growth.

The stronger association between maternal anthropometry and head circumference compared with paternal anthropometry, and the exclusive association between the ‘head and trunk skeletal size’ PC and maternal anthropometry, might reflect processes that serve to prevent a mismatch between fetal size and maternal birth canal dimensions that could otherwise result in obstructed labor (Pembrey, 1996). Maternal height correlates positively with her pelvic dimensions and is an important predictor of obstructed labor (Connolly and McKenna, 2001; Kjærgaard et al., 2010; Benjamin et al., 2012). Furthermore, in the ‘head and trunk skeletal size’ PC, trunk breadths feature relatively prominently along with head size. Given that shoulder dystocia is an important cause of obstructed labor that has been linked to humans’ relatively broad shoulders (Trevathan and Rosenberg, 2000), this may also suggest maternal constraints on fetal head and trunk size to prevent cephalo-pelvic disproportion. Indeed, Veena et al. (2009) reported that in an Indian sample, maternal external pelvic dimensions were an independent predictor of neonatal skeletal head and trunk size, and that maternal height and BMI were much more strongly associated with their neonatal head and trunk PC score than those of the father, consistent with our results and interpretation. Maternal height may thus be associated with overall newborn size due to shared genotype and to prevent obstructed labor.

Maternal BMI indicates aspects of the fetal environment, since maternal BMI may have direct physiological influences on fetal growth through determining, for example, nutrient supply and hormone profiles (King, 2006; Jansson et al., 2008; Higgins et al., 2011). Thus maternal BMI may be associated with neonatal fatness as it reflects maternal resource availability. Increasing neonatal fatness where resources permit may allow the mother to opportunistically enhance early infant growth and survival, with which birthweight and fatness are associated (Karn and Penrose, 1951; Wilcox and Russell, 1983; Wiley, 1994; Kuzawa, 1998).

Paternal anthropometry may be more closely associated with limb size since this enables the father’s (epi)genotype to maximize fetal growth without coming up against strong maternal constraints that act to prevent obstructed labor. Paternal interests may be served by enhancing linear (particularly limb) growth, since this avoids exacerbating obstetric risks while driving greater lean mass accretion. Greater birthweight and length are positively associated with greater adult height and lean mass (Sørensen et al., 1999; Pietiläinen et al., 2002; Eide et al., 2005; Adair, 2007; Wells et al., 2007), and greater height is associated with enhanced reproductive success in both males (Pawlowski et al., 2000; Nettle, 2002; Sear, 2006) and (non-Western) females (Martorell et al., 1981; Sear et al., 2004; Sear, 2006; Pollet and Nettle, 2008). By influencing early lean tissue growth the father may ultimately enhance his offspring’s reproductive success.

As BMI reflects both fat and lean mass, and height is associated with lean mass, the pattern of association between neonatal limb dimensions and paternal anthropometry could reflect a link between paternal lean mass and skeletal size at birth. Previous studies report that paternal height is significantly associated with neonatal fat free mass (Catalano et al., 1995), bone mass (Godfrey et al., 2001), and arm circumference but not skinfolds or birthweight (Knight et al., 2005), suggesting a paternal size effect on neonatal lean mass. Lean mass in fetal life may also then track into adulthood, since associations between birthweight and adult lean mass, but not fat mass, have been documented (Singhal et al., 2003; Sachdev et al., 2005; Wells et al., 2007).

The extent to which relationships between parental and offspring anthropometry are genetic, epigenetic or phenotypic in origin is currently unclear. Epigenetics may play an important role in associations between paternal and offspring metabolism (Kaati et al., 2002; Pembrey, 2002; Lecomte et al., 2013; Wells, 2014), and could also link to prenatal growth. For example, Soubry et al. (2013) recently showed that paternal obesity was associated with hypomethylation of *IGF2*, an important regulator of prenatal growth. The *IGF2* gene is paternally expressed and maternally imprinted in the placenta, and expression during the first trimester is positively associated with offspring birthweight (Demetriou et al., 2014). Thus *IGF2* expression, particularly in early pregnancy, may play a role in early offspring growth, but a number of imprinted loci relating to fetal and neonatal size have been identified in humans that seem to have effects at different times during pregnancy (Hitchins and Moore, 2002; Apostolidou et al., 2007; Ishida et al., 2012; Kumar et al., 2012; Ishida and Moore, 2013; Demetriou et al., 2014). Furthermore the pattern of imprinting may relate to pre-pregnancy and *in utero* environment, as well as offspring sex (Tobi et al., 2009). How maternal and paternal genes are expressed in the growing fetus, and the extent to which their expression is mediated by environmental factors, is a complex area which were are only now beginning to understand.

Previous studies have rarely tested statistically for differences between sons and daughters in the relationship between neonatal and parental phenotype. Though there is suggestive evidence that parental phenotype and prenatal environment might affect the sexes differently (Pembrey et al., 2005; Anderson et al., 2006; Thone-Reineke et al., 2006; Chen et al., 2012; Aiken and Ozanne, 2013), reported patterns are rarely tested statistically. Such differences might be predicted theoretically since males are thought to place a greater demand on maternal physiology (Stinson 1985) due to their faster growth and larger size (Catalano et al., 1995; Hindmarsh et al., 2002; Melamed et al., 2013), and to be more sensitive to early growth disturbance (Stini, 1969; Stinson, 1985; Kuh et al., 1991; Wamani et al., 2007; Ashizawa et al., 2008; Decaro et al., 2010). However, we found no evidence of such a contrast in this study. As the study sample represents a western, relatively wealthy population, whether the same result would be found in more stressful environment where maternal energetics are more marginal remains to be tested.

Parental anthropometry explained a relatively low proportion of variance in neonatal measurements (<7%), indicating the importance of various environmental and genetic factors on both parental and neonatal phenotype. Documented associations between parental and offspring height are stronger in adulthood than at birth, with heritability estimates of around 80% in adulthood for relatively wealthy populations (Silventoinen et al., 2003). Heritability estimates of various measurements including head circumference, height and weight also increase from approximately 6 months of age compared with at birth when they are typically 25-30% (Levine et al., 1987; Demerath et al., 2007; Johnson et al., 2011; Silventoinen et al., 2011; Mook-Kanamori et al., 2012). Thus fetal growth may be generally more sensitive to the environment than postnatal growth, accounting for closer relationships between parental and offspring anthropometry in adulthood. This environmental sensitivity prior to birth may aid in preventing a mismatch between offspring genetic growth potential and maternal body size, which is the outcome of both genetics and past environment, and could raise the risk of obstructed labor (Wells, In press). Various studies of humans and other mammals indicate that maternal size acts to constrain fetal size (Walton and Hammond, 1938; Morton, 1955; Brooks et al., 1995; Wells et al., 2013), presumably to prevent such a mismatch.

The strengths of this study include the large sample size and range of anthropometric and other variables. Many previous studies derive leg length by subtracting crown-rump from crown-heel length, meaning these measurements are not independent and include head size in the total and trunk length measurements. In our dataset, trunk length (neck-rump length), head size and limb lengths were measured independently, permitting their individual associations with parental measurements and their contributions to neonatal anthropometric variation to be more readily separable and interpretable (e.g. Shields et al., 2006; Veena et al., 2009).

Paternal height and the weights of both parents were self reported, so subject to bias (Gorber et al., 2007; McAdams et al., 2007). However, BMI based on self-reported measurements may be sufficiently accurate for epidemiological studies (McAdams et al., 2007). Our analyses also did not include several other factors that have been previously shown to relate to neonatal anthropometry, including maternal pregnancy weight gain (Kramer, 1987; Catalano et al., 1995; Goldenberg et al., 1997; Frederick et al., 2008; Roland et al., 2012; Tikellis et al., 2012), maternal and paternal birthweight (Kramer, 1987; Little, 1987; Emanuel et al., 1992; Magnus et al., 2001), maternal micronutrient status (Kramer, 1987; Mathews et al., 1999; Leffelaar et al., 2010), placental weight (Kramer, 1987; Roland et al., 2012; Tikellis et al., 2012) and maternal glucose metabolism before or during pregnancy (Catalano et al., 2003; HAPO Study Cooperative Research Group, 2009; Catalano et al., 2012; Roland et al., 2012).

Non-paternity may have attenuated associations between paternal and neonatal phenotype. Paternity was not genetically tested in this dataset. Estimated rates of non-paternity in western populations vary from <1% to >30% (Bellis et al., 2005; Anderson, 2006; Voracek et al., 2008). However, the higher estimates derive from studies where participants had already expressed doubt regarding paternity, biasing the samples (Bellis et al., 2005; Anderson, 2006; Voracek et al., 2008). Recent estimates suggest average non-paternity rates of 1-3% in the general population (Bellis et al., 2005; Anderson, 2006; Voracek et al., 2008; Wolf et al., 2012; Larmuseau et al., 2013). Thus given the generally low rates of non-paternity in western populations, this likely had a relatively small influence on our results.

In conclusion, our results suggest that variation in neonatal body measurements may be represented by ‘head and trunk skeletal size’, ‘adiposity’, and ‘limb length’ components, and that different individual measurements or components vary in their associations with parental anthropometry. Paternal body size was particularly associated with limb lengths, while maternal height and BMI were more strongly associated with adiposity and birthweight. We suggest that this may reflect the need to tailor fetal head and trunk size to maternal pelvic dimensions in order to reduce the risk of obstructed labor. Paternal factors may increase maternal physiological investment in the fetus by driving greater limb lengths and lean tissue without exacerbating obstetric risks. While the relationship between neonatal skinfolds and maternal BMI likely reflects an environmental effect on fetal growth while limb and trunk size are more strongly genetically determined, the extent to which parental phenotype mediates head, trunk and limb sizes is unclear. The implications of this study are that neonatal anthropometric phenotype is represented by similar key components across multiple populations, regardless of ethnicity and SES, and that environmental factors, obstetric constraints and parental conflicts of interest may lead to different associations between maternal or paternal body size and distinct components of neonatal phenotype.

**ACKNOWLEDGEMENTS**

The authors thank the MUSP participants, the MUSP Research Team, the MUSP data collection team from Phases 1-2, and the Mater Misericordiae Hospital and the Schools of Social Science, Population Health, and Medicine, at The University of Queensland for their support. We particularly thank Dr Douglas Keeping MB ChB MD FRCOG FRANZCOG, who was responsible for the data collection.

**LITERATURE CITED**

Adair LS. 2007. Size at birth and growth trajectories to young adulthood. Am J Hum Biol 19: 327-337.

Aiken CE, Ozanne SE. 2013. Sex differences in developmental programming models. Reproduction 145:R1-R13.

Albouy-Llaty M, Thiebaugeorges O, Goua V, Magnin G, Schweitzer M, Forhan A, Lelong N, Slama R, Charles MA, Kaminski M et al. . 2011. Influence of fetal and parental factors on intrauterine growth measurements: results of the EDEN mother–child cohort. Ultrasound Obstet Gynecol 38(6):673-680.

Allen W, Wilsher S, Stewart F, Ousey J, Fowden A. 2002a. The influence of maternal size on placental, fetal and postnatal growth in the horse. II. Endocrinology of pregnancy. J Endocrinol 172(2):237-246.

Allen W, Wilsher S, Turnbull C, Stewart F, Ousey J, Rossdale P, Fowden A. 2002b. Influence of maternal size on placental, fetal and postnatal growth in the horse. I. Development in utero. Reproduction 123(3):445-453.

Anderson FJ, Morton S, Naik S, Gebrian B. 2007. Maternal mortality and the consequences on infant and child survival in rural Haiti. Matern Child Health J 11(4):395-401.

Anderson KG. 2006. How well does paternity confidence match actual paternity? Evidence from worldwide nonpaternity rates. Curr Anthropol 47:513-520.

Anderson LM, Riffle L, Wilson R, Travlos GS, Lubomirski MS, Alvord WG. 2006. Preconceptional fasting of fathers alters serum glucose in offspring of mice. Nutrition 22:327-331.

Apostolidou S, Abu-Amero S, O’Donoghue K, Frost J, Olafsdottir O, Chavele KM, Whittaker JC, Loughna P, Stanier P, Moore GE. 2007. Elevated placental expression of the imprinted PHLDA2 gene is associated with low birth weight. J Mol Med 85(4):379-387.

Ashizawa K, Tanamachi N, Kato S, Kumakura C, Zhou XIA, Jin F, Li Y, Lu S. 2008. Growth of height and leg length of children in Beijing and Xilinhot, China. Anthropol Sci 116(1):67-76.

Bailey SM, Xu J, Feng JH, Hu X, Zhang C, Qui S. 2007. Tradeoffs between oxygen and energy in tibial growth at high altitude. Am J Hum Biol 19(5):662-668.

Barker DJP. 1998. Mothers, Babies, and Disease in Later Life. Edinburgh: Churchill Livingstone.

Bellis MA, Hughes K, Hughes S, and Ashton JR. 2005. Measuring paternal discrepancy and its public health consequences. J Epidemiol Community Health 59:749-754.

Benjamin SJ, Daniel AB, Kamath A, Ramkumar V. 2012. Anthropometric measurements as predictors of cephalopelvic disproportion. Acta Obstet Gynecol Scand 91(1):122-127.

Brooks AA, Johnson MR, Steer PJ, Pawson ME, and Abdalla HI. 1995. Birth weight: nature or nurture? Early Hum Dev 42:29-35.

Carone BR, Fauquier L, Habib N, Shea JM, Hart CE, Li R, Bock C, Li C, Gu H, Zamore PD Gu H, Zamore PD, Meissner A, Weng Z, Hofmann HA, Friedman N, Rando OJ. 2010. paternally induced transgenerational environmental reprogramming of metabolic gene expression in mammals. Cell 143:1084-1096.

Catalano PM, Drago NM, Amini SB. 1995. Factors affecting fetal growth and body composition. Am J Obstet Gynecol 172:1459-1463.

Catalano PM, McIntyre HD, Cruickshank JK, McCance DR, Dyer AR, Metzger BE, Lowe LP, Trimble ER, Coustan DR, Hadden DR, Persson B, Hod M, Oats JJN, for the HAPO Study Cooperative Research Group. 2012. The Hyperglycemia and Adverse Pregnancy Outcome Study: Associations of GDM and obesity with pregnancy outcomes. Diabetes Care 35:780-786.

Catalano PM, Thomas A, Huston-Presley L, Amini SB. 2003. Increased fetal adiposity: A very sensitive marker of abnormal in utero development. Am J Obstet Gynecol 189:1698-1704.

Chen Y-P, Xiao X-M, Li J, Reichetzeder C, Wang Z-N, Hocher B. 2012. Paternal Body Mass Index (BMI) is associated with offspring intrauterine growth in a gender dependent manner. PLoS One 7:e36329.

Connolly G, McKenna P. 2001. Maternal height and external pelvimetry to predict cephalo-pelvic disproportion in nulliparous African women. Br J Obstet Gynaecol 108(3):338.

Decaro JA, Decaro E, Worthman CM. 2010. Sex differences in child nutritional and immunological status 5–9 years post contact in fringe highland Papua New Guinea. Am J Hum Biol 22(5):657-666..

Demerath EW, Choh AC, Czerwinski SA, Lee M, Sun SS, Chumlea WMC, Duren D, Sherwood RJ, Blangero J, Towne B et al. 2007. Genetic and environmental influences on infant weight and weight change: The Fels longitudinal study. Am J Hum Biol 19(5):692-702.

Demetriou C, Abu-Amero S, Thomas AC, Ishida M, Aggarwal R, Al-Olabi L, Leon LJ, Stafford JL, Syngelaki A, Peebles D et al. 2014. Paternally expressed, imprinted insulin-like growth factor-2 in chorionic villi correlates significantly with birth weight. PLoS One 9(1):e85454.

Denham M, Schell LM, Gallo M, and Stark A. 2001. Neonatal size of low socio-economic status Black and White term births in Albany County, NYS. Ann Hum Biol 28:172-183.

Dunteman GH. 1989. Principal components analysis. London: Sage.

Eide MG, Øyen N, Skjærven R, Nilsen ST, Bjerkedal T, Tell GS. 2005. Size at birth and gestational age as predictors of adult height and weight. Epidemiol 16: 175-181.

Elshibly EM, Schmalisch G. 2009. Relationship between maternal and newborn anthropometric measurements in Sudan. Pediatr Int 51:326-331.

Emanuel I, Filakti H, Alberman EVA, Evans SJW. 1992. Intergenerational studies of human birthweight from the 1958 birth cohort. 1. Evidence for a multigenerational effect. BJOG 99:67-74.

Frederick I, Williams M, Sales A, Martin D, Killien M. 2008. Pre-pregnancy body mass index, gestational weight gain, and other maternal characteristics in relation to infant birth weight. Matern Child Health J 12:557-567.

Godfrey K, Walker-Bone K, Robinson S, Taylor P, Shore S, Wheeler T, Cooper C. 2001. Neonatal bone mass: influence of parental birthweight, maternal smoking, body composition, and activity during pregnancy. J Bone Mineral Res 16:1694-1703.

Godfrey KM, Barker DJP, Robinson S, Osmond C. 1997. Maternal birthweight and diet in pregnancy in relation to the infant's thinness at birth. BJOG 104:663-667.

Goldenberg RL, Cliver SP, Neggers Y, Copper RL, DuBard MD, Davis RO, Hoffman HJ. 1997. The relationship between maternal characteristics and fetal and neonatal anthropometric measurements in women delivering at term: a summary. Acta Obstet Gynecol Scand Supplement 165:8-13.

Gorber SC, Tremblay M, Moher D, Gorber B. 2007. A comparison of direct vs. self-report measures for assessing height, weight and body mass index: a systematic review. Obesity Reviews 8:307-326.

Griffiths LJ, Dezateux C, Cole TJ, Millennium Cohort Study Child Health Group. 2007. Differential parental weight and height contributions to offspring birthweight and weight gain in infancy. Int J Epidemiol 36:104-107.

Haig D, Westoby M. 1989. Parent-specific gene expression and the triploid endosperm. Am Nat 134(1):147-155.

Hales CN, Barker DJ. 1992. Type 2 (non-insulin-dependent) diabetes mellitus: the thrifty phenotype hypothesis. Diabetologia 35:595-601.

HAPO Study Cooperative Research Group. 2009. Hyperglycemia and Adverse Pregnancy Outcome (HAPO) Study: associations with neonatal anthropometrics. Diabetes 58:453-459.

Harvey NC, Poole JR, Javaid MK, Dennison EM, Robinson S, Inskip HM, Godfrey KM, Cooper C, Sayer AA, and and the SWS Study Group. 2007. Parental determinants of neonatal body composition. J Clin Endocrinol Metabol 92:523-526.

Higgins L, Greenwood SL, Wareing M, Sibley CP, Mills TA. 2011. Obesity and the placenta: A consideration of nutrient exchange mechanisms in relation to aberrant fetal growth. Placenta 32:1-7.

Hillman S, Peebles DM, Williams DJ. 2013. Paternal metabolic and cardiovascular risk factors for fetal growth restriction: A case-control study. Diabetes Care 36:1675-1680.

Hindmarsh PC, Geary MP, Rodeck CH, Kingdom JC, Cole TJ. 2002. Intrauterine growth and its relationship to size and shape at birth. Pediatr Res 52:263-268.

Hitchins MP, Moore GE. 2002. Genomic imprinting in fetal growth and development. Expert Rev Mol Med 4: 1-19.

Hyppönen E, Smith GD, Power C. 2003. Parental diabetes and birth weight of offspring: Intergenerational cohort study. BMJ 326:19-20.

Ishida M, Monk D, Duncan Andrew J, Abu-Amero S, Chong J, Ring Susan M, Pembrey Marcus E, Hindmarsh Peter C, Whittaker John C, Stanier P et al. . 2012. Maternal Inheritance of a promoter variant in the imprinted PHLDA2 gene significantly increases birth weight. Am J Hum Genet 90: 715-719.

Ishida M, Moore GE. 2013. The role of imprinted genes in humans. Mol Aspects Med 34: 826-840.

Jansen PW, Tiemeier H, Looman CWN, Jaddoe VWV, Hofman A, Moll HA, Steegers EAP, Verhulst FC, Mackenbach JP, Raat H. 2009. Explaining educational inequalities in birthweight: the Generation R Study. Paediatr Perinat Epidemiol 23:216-228.

Jansson N, Nilsfelt A, Gellerstedt M, Wennergren M, Rossander-Hulthén L, Powell TL, Jansson T. 2008. Maternal hormones linking maternal body mass index and dietary intake to birth weight. Am J Clin Nutr 87:1743-1749.

Johnson L, Llewellyn CH, van Jaarsveld CHM, Cole TJ, and Wardle J. 2011. Genetic and environmental influences on infant growth: Prospective analysis of the Gemini Twin Birth Cohort. PLoS One 6(5):e19918

Kaati G, Bygren LO, and Edvinsson S. 2002. Cardiovascular and diabetes mortality determined by nutrition during parents' and grandparents' slow growth period. Eur J Hum Genet 10:682-688.

Kaiser H. 1958. The varimax criterion for analytic rotation in factor analysis. Psychometrika 23(3):187-200.

Karn MN, Penrose LS. 1951. Birth weight and gestation time in relation to maternal age, parity and infant survival. Ann Eugen 16(1):147-164.

Keeping JD. 1981. Determinants and components of size. Unpublished PhD Thesis: University of Aberdeen.

King JC. 2006. Maternal obesity, metabolism, and pregnancy outcomes. Annu Rev Nutr 26:271-291.

Kjærgaard H, Dykes AK, Ottesen B, Olsen J. 2010. Risk indicators for dystocia in low-risk nulliparous women: A study on lifestyle and anthropometrical factors. J Obstet Gynaecol 30(1):25-29.

Knight B, Shields BM, Turner M, Powell RJ, Yajnik CS, Hattersley AT. 2005. Evidence of genetic regulation of fetal longitudinal growth. Early Hum Dev 81:823-831.

Koyanagi A, Zhang J, Dagvadorj A, Hirayama F, Shibuya K, Souza JP, Gülmezoglu AM. 2013. Macrosomia in 23 developing countries: an analysis of a multicountry, facility-based, cross-sectional survey. Lancet 381(9865):476-483.

Kramer MS. 1987. Determinants of low birth weight: methodological assessment and meta-analysis. Bull World Health Organ 65:663-737.

Kramer MS, Séguin L, Lydon J, Goulet L. 2000. Socio-economic disparities in pregnancy outcome: why do the poor fare so poorly? Paediatr Perinat Epidemiol 14:194-210.

Kuh DL, Power C, Rodgers B. 1991. Secular trends in social class and sex differences in adult height. Int J Epidemiol 20(4):1001-1009.

Kumar N, Leverence J, Bick D, Sampath V. 2012. Ontogeny of growth-regulating genes in the placenta. Placenta 33(2):94-99.

Kuzawa C. 1998. Adipose tissue in human infancy and childhood: an evolutionary perspective. Yrbk Phys Anthropol 41: 177-209.

Kuzawa CW, and Eisenberg DTA. 2012. Intergenerational predictors of birth weight in the Philippines: Correlations with mother’s and father’s birth weight and test of maternal constraint. PLoS One 7:e40905.

Lampl M, Kuzawa CW, Jeanty P. 2003. Prenatal smoke exposure alters growth in limb proportions and head shape in the midgestation human fetus. Am J Hum Biol 15(4):533-546.

Larmuseau MHD, Vanoverbeke J, Van Geystelen A, Defraene G, Vanderheyden N, Matthys K, Wenseleers T, and Decorte R. 2013. Low historical rates of cuckoldry in a Western European human population traced by Y-chromosome and genealogical data. Proc R Soc Lond B Biol Sci 280(1772): 20132400.

Leary S, Fall C, Osmond C, Lovel H, Campbell D, Eriksson J, Forrester T, Godfrey K, Hill J, Jie M, Law C, Newby R, Robinson S, Yajnik C. 2006. Geographical variation in relationships between parental body size and offspring phenotype at birth. Acta Obstet Gynecol Scand 85:1066-1079.

Lecomte V, Youngson NA, Maloney CA, Morris MJ. 2013. Parental programming: How can we improve study design to discern the molecular mechanisms? BioEssays 35:787-793.

Leffelaar ER, Vrijkotte TGM, and van Eijsden M. 2010. Maternal early pregnancy vitamin D status in relation to fetal and neonatal growth: results of the multi-ethnic Amsterdam Born Children and their Development cohort. Br J Nutrn 104:108-117.

Levine RS, Hennekens CH, and Jesse MJ. 1987. Genetic variance of weight and length in infant twins. Am J Epidemiol 126(5):929-935.

Lindsay RS, Dabelea D, Roumain J, Hanson RL, Bennett PH, Knowler WC. 2000. Type 2 diabetes and low birth weight: the role of paternal inheritance in the association of low birth weight and diabetes. Diabetes 49:445-449.

Liselele HB, Boulvain M, Tshibangu KC, Meuris S. 2000. Maternal height and external pelvimetry to predict cephalopelvic disproportion in nulliparous African women: a cohort study. Br J Obstet Gynaecol 107(8):947-952.

Little RE. 1987. Mother's and father's birthweight as predictors of infant birthweight. Paediatr Perinat Epidemiol 1:19-31.

Lummaa V. 2003. Early Developmental conditions and reproductive success in humans: Downstream effects of prenatal famine, birthweight, and timing of birth. Am J Hum Biol 15: 370-379.

Magnus P, Gjessing HK, Skrondal A, Skjærven R. 2001. Paternal contribution to birth weight. J Epidemiol Community Health 55:873-877.

Martorell R, Delgado HL, Delgado H, Valverde V, Klein RE. 1981. Maternal stature, fertility and infant mortality. Hum Biol 53: 303-312.

Mathews F, Yudkin P, Neil A. 1999. Influence of maternal nutrition on outcome of pregnancy: prospective cohort study. BMJ 319:339.

McAdams MA, Van Dam RM, Hu FB. 2007. Comparison of self-reported and measured BMI as correlates of disease markers in US adults. Obesity (Silver Spring) 15:188-196.

McGrath JJ, Keeping D, Saha S, Chant DC, Lieberman DE, O'Callaghan MJ. 2005. Seasonal fluctuations in birth weight and neonatal limb length; does prenatal vitamin D influence neonatal size and shape? Early Hum Dev 81:609-618.

McIntire DD, Bloom SL, Casey BM, Leveno KJ. 1999. Birth Weight in Relation to Morbidity and Mortality among Newborn Infants. New Engl J Med 340(16):1234-1238.

Meadows Jantz L, Jantz RL. 1999. Secular change in long bone length and proportion in the United States, 1800-1970. Am J Phys Anthropol 110(1):57-67.

Mei Z, Grummer-Strawn LM, Thompson D, Dietz WH. 2004. Shifts in percentiles of growth during early childhood: Analysis of longitudinal data from the California Child Health and Development Study. Pediatr 113(6):e617-627.

Melamed N, Meizner I, Mashiach R, Wiznitzer A, Glezerman M, Yogev Y. 2013. Fetal Sex and Intrauterine Growth Patterns. J Ultrasound Med 32(1):35-43

Mook-Kanamori DO, van Beijsterveldt CEM, Steegers EAP, Aulchenko YS, Raat H, Hofman A, Eilers PH, Boomsma DI, and Jaddoe VWV. 2012. Heritability estimates of body size in fetal life and early childhood. PLoS One 7(7):e39901.

Moore T, Haig D. 1991. Genomic imprinting in mammalian development: a parental tug-of-war. Trends Genet 7(2):45-49.

Morrison J, Williams GM, Najman JM, Andersen MJ. 1991. The influence of paternal height and weight on birth-weight. Aust N Z J Obstet Gynaecol 31:114 –116.

Morton BNE. 1955. The inheritance of human birth weight. Ann Hum Genet 20:125-134.

Myklestad K, Vatten LJ, Magnussen EB, Salvesen KÅ, Smith GD, Romundstad PR. 2012. Offspring birth weight and cardiovascular risk in parents - A population-based HUNT 2 Study. Am J Epidemiol 175:546-555.

Najman J, Bor W, O'Callaghan M, Williams G, Aird R, Shuttlewood G. 2005. Cohort Profile: The Mater-University of Queensland Study of Pregnancy (MUSP). Int J Epidemiol 34:992-997.

Nettle D. 2002. Height and reproductive success in a cohort of British men. Hum Nat 13: 473-491.

Ng S-F, Lin RCY, Laybutt DR, Barres R, Owens JA, Morris MJ. 2010. Chronic high-fat diet in fathers programs β-cell dysfunction in female rat offspring. Nature 467:963-966.

Pawlowski B, Dunbar RIM, Lipowicz A. 2000. Tall men have more reproductive success. Nature 403(6766): 156-156.

Pembrey M. 1996. Imprinting and transgenerational modulation of gene expression; human growth as a model. Acta Genet Med Gemellol (Roma) 45: 111-125.

Pembrey ME. 2002. Time to take epigenetic inheritance seriously. Eur J Hum Genet 10(11):669-671.

Pembrey ME, Bygren LO, Kaati G, Edvinsson S, Northstone K, Sjostrom M, Golding J. 2005. Sex-specific, male-line transgenerational responses in humans. Eur J Hum Genet 14:159-166.

Pietiläinen KH, Kaprio J, Räsänen M, Rissanen A, Rose RJ. 2002. Genetic and environmental influences on the tracking of body size from birth to early adulthood. Obes Res 10: 875-884.

Pollet TV, Nettle D. 2008. Taller women do better in a stressed environment: Height and reproductive success in rural Guatemalan women. Am J Hum Biol 20: 264-269.

Pomeroy E, Stock JT, Stanojevic S, Miranda JJ, Cole TJ, Wells JCK. 2012. Trade-offs in relative limb length among Peruvian children: Extending the thrifty phenotype hypothesis to limb proportions. PLoS One 7:e51795.

Pomeroy E, Stock JT, Stanojevic S, Miranda JJ, Cole TJ, Wells JCK. 2013. Associations between arterial oxygen saturation, body size and limb measurements among high-altitude Andean children. Am J Hum Biol 25(5):629-636.

Pomeroy E, Wells JCK, Stock JT. In press. Obstructed labour: the classic obstetric dilemma and beyond. In: Alvergne A, Faurie C, and Jenkinson C, editors. *Evolutionary thinking in medicine: from research to policy and practice*. Springer.

Raum E, Arabin B, Schlaud M, Walter U, Schwartz FW. 2001. The impact of maternal education on intrauterine growth: a comparison of former West and East Germany. Int J Epidemiol 30:81-87.

Roland MCP, Friis CM, Voldner N, Godang K, Bollerslev J, Haugen G, Henriksen T. 2012. Fetal growth versus birthweight: The role of placenta versus other determinants. PLoS One 7:e39324.

Rosenberg K, Trevathan W. 2002. Birth, obstetrics and human evolution. Br J Obstet Gynaecol 109(11):1199-1206.

Sachdev HS, Fall CH, Osmond C, Lakshmy R, Dey Biswas SK, Leary SD, Reddy KS, Barker DJ, Bhargava SK. 2005. Anthropometric indicators of body composition in young adults: relation to size at birth and serial measurements of body mass index in childhood in the New Delhi birth cohort. Am J Clin Nutr 82: 456-466.

Sear R. 2006. Height and reproductive success. Hum Nat 17: 405-418.

Sear R, Allal N, Mace R. 2004. Height, marriage and reproductive success in Gambian women. Res Econ Anthropol 23: 203-224.

Shields B, Knight B, Powell R, Hattersley A, Wright D. 2006. Assessing newborn body composition using principal components analysis: differences in the determinants of fat and skeletal size. BMC Pediatr 6:24.

Silventoinen K, Karvonen M, Sugimoto M, Kaprio J, Dunkel L, and Yokoyama Y. 2011. Genetics of head circumference in infancy: A longitudinal study of Japanese twins. Am J Hum Biol 23(5):630-634.

Silventoinen K, Sammalisto S, Perola M, Boomsma DI, Cornes BK, Davis C, Dunkel L, de Lange M, Harris JR, Hjelmborg JVB et al. . 2003. Heritability of Adult Body Height: A Comparative Study of Twin Cohorts in Eight Countries. Twin Res 6(5):399-408.

Singhal A, Wells J, Cole TJ, Fewtrell M, Lucas A. 2003. Programming of lean body mass: a link between birth weight, obesity, and cardiovascular disease? Am J Clin Nutr 77: 726-730.

Sletner L, Nakstad B, Yajnik CS, Mørkrid K, Vangen S, Vårdal MH, Holme IM, Birkeland KI, Jenum AK. 2013. Ethnic differences in neonatal body composition in a multi-ethnic population and the impact of parental factors: A population-based cohort study. PLoS One 8(8):e73058.

Smith DW, Truog W, Rogers JE, Greitzer LJ, Skinner AL, McCann JJ, Sedgwick Harvey MA. 1976. Shifting linear growth during infancy: Illustration of genetic factors in growth from fetal life through infancy. J Pediatr 89(2):225-230.

Sørensen HT, Sabroe S, Rothman KJ, Gillman M, Steffensen FH, Fischer P, Serensen TIA. 1999. Birth weight and length as predictors for adult height. Am J Epidemiol 149: 726-729.

Soubry A, Schildkraut J, Murtha A, Wang F, Huang Z, Bernal A, Kurtzberg J, Jirtle R, Murphy S, Hoyo C. 2013. Paternal obesity is associated with IGF2 hypomethylation in newborns: results from a Newborn Epigenetics Study (NEST) cohort. BMC Medicine 11:29.

Stini WA. 1969. Nutritional stress and growth: Sex difference in adaptive response. Am J Phys Anthropol 31(3):417-426.

Stinson S. 1985. Sex differences in environmental sensitivity during growth and development. Am J Phys Anthropol 28(S6):123-147.

Thone-Reineke C, Kalk P, Dorn M, Klaus S, Simon K, Pfab T, Godes M, Persson P, Unger T, Hocher B. 2006. High-protein nutrition during pregnancy and lactation programs blood pressure, food efficiency, and body weight of the offspring in a sex-dependent manner. Am J Physiol Regul Integr Comp Physiol 291:R1025-1030.

Tikellis G, Ponsonby AL, Wells JCK, Pezic A, Cochrane J, Dwyer T. 2012. Maternal and infant factors associated with neonatal adiposity: Results from the Tasmanian Infant Health Survey (TIHS). Int J Obes 36:496-504.

To WWK, Cheung W, Kwok JSY. 1998. Paternal height and weight as determinants of birth weight in a Chinese population. Amer J Perinatol 15:545-548.

Tobi EW, Lumey LH, Talens RP, Kremer D, Putter H, Stein AD, Slagboom PE, Heijmans BT. 2009. DNA methylation differences after exposure to prenatal famine are common and timing- and sex-specific. Hum Mol Genet 18(21):4046-4053.

Trevathan W, Rosenberg K. 2000. The shoulders follow the head: postcranial constraints on human childbirth. J Hum Evol 39(6):583-586.

Tyrrell JS, Yaghootkar H, Freathy RM, Hattersley AT, Frayling TM. 2013. Parental diabetes and birthweight in 236 030 individuals in the UK Biobank Study. Int J Epidemiol 42:1714-1723.

van den Berg G, van Eijsden M, Galindo-Garre F, Vrijkotte TGM, Gemke RJBJ. 2013. Smoking overrules many other risk factors for small for gestational age birth in less educated mothers. Early Hum Dev 89:497-501.

Veena S, Krishnaveni G, Wills A, Hill J, Fall C. 2009. A principal components approach to parent-to-newborn body composition associations in South India. BMC Pediatrics 9:16.

Villar J, Ismail LC, Victora CG, Ohuma EO, Bertino E, Altman DG, Lambert A, Papageorghiou AT, Carvalho M, Jaffer YA et al. 2014. International standards for newborn weight, length, and head circumference by gestational age and sex: the Newborn Cross-Sectional Study of the INTERGROWTH-21st Project. Lancet 384(9946):857-868.

Voigt M, Heineck G, Hesse V. 2004. The relationship between maternal characteristics, birth weight and pre-term delivery: evidence from Germany at the end of the 20th century. Econ Hum Biol 2:265-280.

Voracek M, Haubner T, and Fisher ML. 2008. Recent decline in nonpaternity rates: A cross-temporal meta-analysis. Psychol Rep 103(3):799-811.

Walton A, and Hammond J. 1938. The maternal effects on growth and conformation in shire horse-Shetland pony crosses. Proc R Soc Lond B Biol Sci 125(840):311-335.

Wamani H, Astrom A, Peterson S, Tumwine J, Tylleskar T. 2007. Boys are more stunted than girls in Sub-Saharan Africa: a meta-analysis of 16 demographic and health surveys. BMC Pediatr 7(1):17.

Wei Y, Yang C-R, Wei Y-P, Zhao Z-A, Hou Y, Schatten H, and Sun Q-Y. 2014. Paternally induced transgenerational inheritance of susceptibility to diabetes in mammals. Proc Natl Acad Sci U S A 111(5):1873-1878.

Wells, JCK. 2014. Paternal and maternal influences on offspring phenotype: The same, only different. Int J Epidemiol 43(3): 772-774.

Wells, JCK. In press. Between Scylla and Charybdis: Re-negotiating the ‘obstetric dilemma’ in response to ecological change. Philos Trans R Soc Lond B Biol Sci.

Wells JCK, Chomtho S, Fewtrell MS. 2007. Programming of body composition by early growth and nutrition. Proc Nutr Soc 66: 423-434.

Wells JCK, Sharp G, Steer PJ, Leon DA. 2013. Paternal and maternal influences on differences in birth weight between Europeans and Indians born in the UK. PLoS One 8:e61116.

Wilcox AJ, Russell IT. 1983. Birthweight and perinatal mortality: II. On weight-specific mortality. Int J Epidemiol 12: 319-325.

Wiley AS. 1994. Neonatal size and infant mortality at high altitude in the western Himalaya. Am J Phys Anthropol 94: 289-305.

Willison K. 1991. Opposite imprinting of the mouse Igf2 and Igf2r genes. Trends Genet 7(4):107-109.

Wolf M, Musch J, Enczmann J, Fischer J. 2012. Estimating the prevalence of nonpaternity in Germany. Hum Nat 23(2):208-217.

**FIGURE LEGENDS**

**Fig 1. Flow chart describing composition of the study sample and its relationship to the full Mater-University of Queensland Study of Pregnancy (MUSP) dataset**

**Fig. 2. Adjusted R² values for variation in neonatal anthropometry explained by parental anthropometry. a) Absolute measurements; b) limb proportion indices**

**Fig. 3. Standardized coefficients (β) for variation in neonatal anthropometry explained by parental anthropometry**
